# Interleukin-1 Receptor-Associated Kinase 2- and Protein Kinase D1-Dependent Regulation of IRAK-Monocyte Expression by CpG DNA

**DOI:** 10.1371/journal.pone.0043970

**Published:** 2012-08-23

**Authors:** Young-In Kim, Jeoung-Eun Park, Ki Han Kwon, Cheol Yi Hong, Ae-Kyung Yi

**Affiliations:** 1 Children's Foundation Research Institute at Le Bonheur Children's Hospital and Department of Pediatrics, University of Tennessee Health Science Center, Memphis, Tennessee, United States of America; 2 Specialized Research Center for Cancer Immunotherapy, Chonnam National University, Jeonnam, Korea; 3 Department of Microbiology, Immunology and Biochemistry, University of Tennessee Health Science Center, Memphis, Tennessee, United States of America; Louisiana State University, United States of America

## Abstract

As a part of the negative feedback mechanism, CpG DNA induces IRAK-M expression in monocytic cells. In the present study we investigated a biochemical signaling pathway and the transcription factors responsible for CpG DNA-mediated *Irak-m* gene expression. CpG DNA-induced *Irak-m* expression did not require new protein synthesis and was regulated at the transcriptional level through an endosomal pH-sensitive TLR9/MyD88 signaling pathway. Over-expression of the dominant negative (DN) form of or gene-specific knockdown of signaling modulators in the TLR9 pathway demonstrated that IRAK4, IRAK1, IRAK2, and PKD1 are required for *Irak-m* transcription induced by CpG DNA. Over-expression of DN-IRAK1 only partially, but significantly, inhibited CpG DNA-induced *Irak-m* promoter activity. While IRAK1 was critical for the initial phase, IRAK2 was required for the late phase of TLR9 signaling by sustaining activation of PKD1 that leads to activation of NF-κB and MAPKs. *Irak-m* promoter-luciferase reporters with alterations in the predicted *cis*-acting transcriptional regulatory elements revealed that the NF-κB consensus site in the *Irak-m* promoter region is absolutely required for *Irak-m* gene expression. AP-1 and CREB binding sites also contributed to the optimal *Irak-m* expression by CpG DNA. Collectively, our results demonstrate that IRAK2 plays a key role in the TLR9-mediated transcriptional regulation of *Irak-m* expression by sustaining activation of PKD1 and NF-κB.

## Introduction

Detection of unique molecular structures of microbial origin (called pathogen-associated molecular patterns; PAMPs) by pattern recognition receptors, such as Toll-like receptors (TLRs), expressed in immune cells is key to activation of the innate host defense mechanisms. Among the various PAMPs, bacterial DNA, double stranded viral DNA, and synthetic oligodeoxynucleotides containing an unmethylated CpG motif (CpG DNA) bind to TLR9 [1]. Upon recognition of its ligand, TLR9 recruits a Toll/IL-1-receptor homology (TIR) domain-containing adaptor protein, myeloid differentiation protein 88 (MyD88) [2], [3]. The binding of MyD88 to TLR9 leads to the subsequent recruitment of interleukin-1 receptor-associated kinase (IRAK) family members, IRAK4 and IRAK1 [4], [5], [6]. IRAK1 becomes rapidly phosphorylated by IRAK4, resulting in recruitment of TNF receptor-associated factor 6 (TRAF6) to the receptor complex [4], [7]. Phosphorylated IRAK1 and TRAF6 are thought to dissociate from the receptor complex, which is followed by TRAF6 autoubiquitination with K63-linked polyubiquitin chains and subsequent polyubiquitination of IRAK1 by TRAF6 [8], [9]. While ubiquitinated IRAK1 is degraded in the proteosome, ubiquitinated TRAF6 binds to and activates a signaling complex composed of TGFβ-activated kinase 1 (TAK1) and TAK1-binding protein 2 (TAB2) [10], [11]. Activation of TAK1 initiates signaling cascades that lead to activation of NF-κB and mitogen-activated protein kinases (MAPKs), and subsequent expression of proinflammatory cytokines and chemokines [12], [13], [14], [15]. In addition to this well-known TLR9 signaling pathway, recent studies provide evidence that IRAK2 interacts with IRAK4 and TRAF6 in the absence of IRAK1, is activated by IRAK4, and plays a critical role in sustaining activation of NF-κB and p38 and expression of proinflammatory genes induced by various TLR ligands, including the ligand for TLR9 [16], [17]. In addition, we recently found that a serine/threonine kinase, protein kinase D1 (PKD1), is recruited to and activated in the TLR9/MyD88 receptor complex *via* an interaction with IRAK4, IRAK1 and TRAF6 [18], [19]. While its activation by CpG DNA is dependent on MyD88, IRAK4, and IRAK1, PKD1 is required for ubiquitination of TRAF6 and subsequent activation of TAK1, MAPKs, and NF-κB, as well as expression of proinflammatory genes [18].

Although innate inflammatory responses induced by ligands for TLR9 and other TLRs are essential for the eradication of infectious microorganisms, excessive and prolonged activation of innate immunity is detrimental to the host. As a part of negative regulatory mechanisms to prevent exaggerated inflammatory reactions, TLR ligands induce several negative regulators, such as IL-10, Src homology 2 domain-containing inositol polyphosphate phosphatase 1, suppressor of cytokine signaling proteins, and IRAK-monocyte (IRAK-M) [20], [21], [22], [23], [24], [25]. Among these, IRAK-M has been demonstrated to down-regulate the inflammatory response by directly blocking TLR/MyD88 signal transduction [20]. IRAK-M (also known as IRAK3) is one of four IRAK family members. Unlike other IRAK family proteins that are active kinases and are expressed ubiquitously and constitutively [4], [27], IRAK-M is catalytically inactive and its expression is induced by various TLR ligands [26], [28], [29], [30]. IRAK-M binds to both IRAK1 and IRAK4 and prevents dissociation of these kinases from the TLR/MyD88 complexes. As a consequence, IRAK-M blocks association of IRAK1 and TRAF6, the step that is required for activation of downstream signaling cascades that eventually lead to inflammatory gene expression [20]. Macrophages hyporesponsive to TLR ligands express high levels of IRAK-M [28], [29], [30]. Furthermore, macrophage hyporesponsiveness induced after the first exposure to LPS is not observed in *Irak-m* ^−/−^ macrophages, indicating an essential role for IRAK-M in the development of endotoxin tolerance [20]. These findings suggest that IRAK-M expression induced by TLR ligand stimulation is one of the central negative feedback mechanisms for the TLR-initiated innate immune response. Studies with IRAK-M-deficient (*Irak-m* ^−/−^) mice confirmed IRAK-M as a negative regulator of TLR/IL-1R signaling [20]. Although the physiologic role and action mechanism of IRAK-M have been uncovered, the biochemical mechanisms by which TLR ligands induce expression of IRAK-M are yet to be understood. In the present study we investigated biochemical signaling pathways and the transcription factors responsible for transcriptional regulation of *Irak-m* expression induced by a TLR9 ligand, CpG DNA.

**Table 1:** *Cis*-acting element consensus sequences changed by site-directed mutagenesis.

Gene		Sequence
NF-κB (2) (−1098/−1089)	Wild type	GGCGATTTCC
	Mutant	AATAGCCCTT
AP-1 (−820/−814)	Wild type	TGAAACA
	Mutant	CAGGTTG
NF-κB (1) (−336/−326)	Wild type	GGCGGCTTTCC
	Mutant	AATAATCCCTT
CREB (−138/−130)	Wild type	CCTACGTCA
	Mutant	TTCGTACTG

## Materials and Methods

### Oligodeoxynucleotides and reagents

Nuclease-resistant phosphorothioate oligodeoxynucleotides (S-ODN) 1826 (CpG DNA) and 1982 (non-CpG DNA) were purchased from Operon (Alameda, CA) and Coley Pharmaceutical Group (Kanata, ON, Canada) and further purified by ethanol precipitation. S-ODN had no detectable endotoxins by *Limulus* assay. The sequences of S-ODN used have been previously reported [31]. Cycloheximide (CHX) and chloroquine were purchased from Sigma Chemical Co. (St. Louis, MO). Ultra pure lipopolysaccharide (LPS; from *Escherichia coli* 0111:B4) was purchased from List Biological Laboratories, Inc. (Campbell, CA). IFNγ was purchased from BD Biosciences (San Jose, CA).

**Table 2:** Sequences of ODN probes for EMSA.

Gene	Sequence
IRAK-M NF-κB(2) Wild type	ACCACACATGGCGATTTCCTGTGCAGGC
IRAK-M NF-κB(2) Mutant	ACCACACATGGCTGTGTATTGTACAGGC
IRAK-M AP1 Wild type	CTCAGGGGATTGAAACAGGAGTTTGTTTTTCAG

### Generation of gene-specific knockdown macrophages, cell lines, and culture conditions

Generation of *Caenorhabditis elegans* luciferase- and mouse protein kinase D1 gene (*Prkd1*)-knockdown macrophages using a vector expressing a gene-specific small hairpin interfering (sh) RNA under H1 promoter was previously described [19]. For generation of control and *Irak2*-knockdown macrophages, RAW264.7 cells (ATCC, Rockville, MD) were plated at 2.5×10^5^ cells/500 μl in a 24-well plate and incubated overnight, then transfected with 100 nM non-target small interfering (si) RNA (Dharmacon, Lafayette, CO) or a mixture of *Irak2*-specific siRNAs (*Irak2-*siRNA 595: 5′GCAGAUGUCGUCCAAGCAAUU3′ and *Irak2-*siRNA 1183: 5′GACAUCUUCAGCUGUGGAAUU3′), respectively, using Lipofectamine (Invitrogen, Carlsbad, CA) according to the manufacturer's protocol. All cells were maintained in DMEM supplemented with 10% (v/v) heat-inactivated fetal bovine serum, 1.5 mM L-glutamine, 100 U/ml penicillin, and 100 μg/ml streptomycin and cultured at 37°C in a 5% CO_2_ humidified incubator. All culture reagents were purchased from Life Technologies (Gaithersburg, MD).

**Table 3:** Sequences of primers for ChIP assay.

Binding site	Forward	Reverse
IRAK-M promoter [NF-κB(2) binding region]	GCTCAGCATGGTGGCATAGAGAC	GGAAACATTGGCTGGTGTGTTGA
IRAK-M promoter (AP-1 binding region)	AGAGGTCCCGGTGAGTTAACATGG	CACAAGACCCAGGAGAGGTCTATG
IRAK-M promoter (CREB binding region)	AAACGGAGACCGAGGGAGCCTTAC	TCTGGGCAGCCTCAGTCCTATTAC
IRAK-M promoter 3′ End	CCGCCAGACAGAACTAGCCGAAC	GTGTACAGGACAAGTGCCAAAG

### Plasmids

To construct the *Irak-m* promoter reporter, 1952 base pairs (−1898/+54; translation start site assigned as +1) in the 5′ region of the *Irak-m* gene were amplified by polymerase chain reaction (PCR) using mouse genomic DNA as a template. The resulting PCR product was cloned into Tez vector (Promega, Madison, WI) to generate the *Irak-m* promoter template (Tez-IRAK-MP). Using IRAK-MP-L2 primer (5′AGACCTGGTGATGCAAACAT3′) and IRAK-MP-R NcoI primer (5′ACAGCGCGCCATGGGCCCGCACC3′), which contains an NcoI restriction site, and Tez-IRAK-MP as a template, 1527 base pairs (−1493/+34) in the 5′ region of the *Irak-m* gene were amplified by PCR. The resulting PCR product was digested with KpnI and NcoI. The resulting KpnI x NcoI fragment containing the *Irak-m* promoter region (−1315/+10) was subcloned into the KpnI and NcoI sites of the pGL3 basic luciferase expression vector (Promega) to generate a wild type *Irak-m*-promoter luciferase-based reporter gene (*Irak-m*-promoter-luc). *Cis-*acting element response sites in the *Irak-m* promoter region were identified by sequence analysis using the TRASFAC v6.0 soft ware (www.upenn.edu/cgi-bin/tess/tess). Putative *cis*-acting element response sites in the *Irak-m* promoter are NF-κB (2) (−1098), AP1 (−820), NF-κB (1) (−336), and CREB (−138). Deletion mutants of the *Irak-m* promoter were generated by PCR using the wild type *Irak-m* promoter-luciferase reporter construct as a template. Each of the resulting *Irak-m* promoter deletion fragments, Δ-1086 (−1086/+10; lacks distal NF-κB site), Δ−756 (−756/+10; lacks distal NF-κB and AP-1 sites), Δ-406 (−406/+10; lacks distal NF-κB and AP-1 sites), Δ-215 (−211/+10; lacks distal NF-κB, AP-1 sites and proximal NF-κB), or Δ-49 (−49/+10; lacks distal NF-κB, AP-1, proximal NF-κB and CREB sites), were cloned into the pGL3 basic luciferase expression vector to generate deletion mutant *Irak-m* promoter-luciferase reporters. Site-directed mutagenesis was performed to modify each *cis*-acting element response site in the *Irak-m* promoter region of the wild type *Irak-m* promoter-luciferase reporter using the QuickChange mutagenesis kit (Stratagene, La Jolla, CA) according to the manufacturer's protocol.

**Figure 1:**
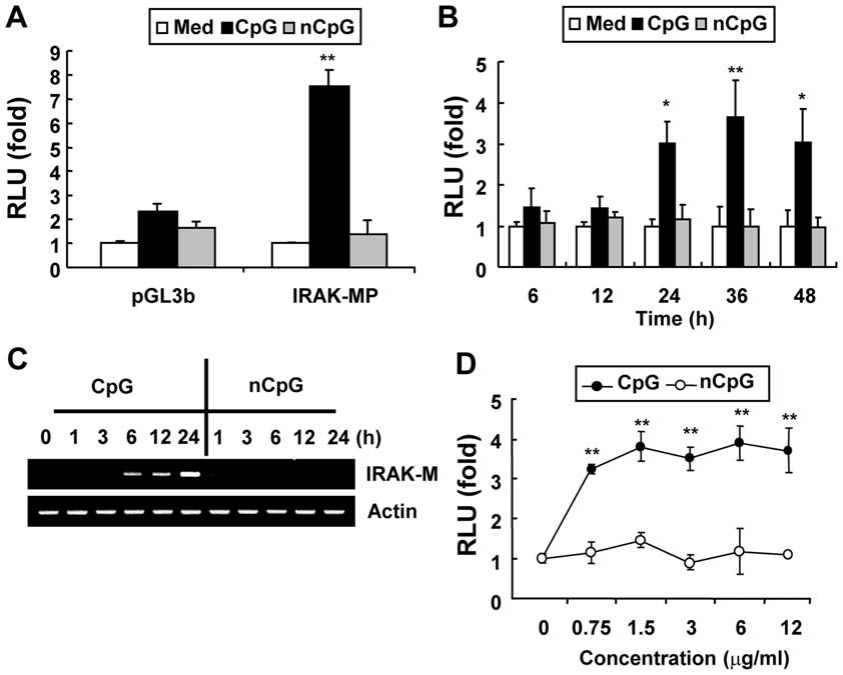
CpG DNA up-regulates *Irak-m* promoter activity. **Panels A, B and D.** RAW264.7 cells were transiently cotransfected with pGL3 basic luciferase (control vector) or *Irak-m*-promoter-luciferase and pRL-TK-luciferase reporters. (**A**) Cells were stimulated with medium, CpG DNA (6 μg/ml), or non-CpG DNA (6 μg/ml) for 24 hr. (**B**) Cells were stimulated with medium, CpG DNA (6 μg/ml), or non-CpG DNA (6 μg/ml) for the indicated time periods. (**D**) Cells were stimulated with medium, CpG DNA (the indicated concentration), or non-CpG DNA (the indicated concentration) for 24 hr. Luciferase activity in cell extracts was analyzed by the Dual-Luciferase Reporter Assay System and normalized using pRL-TK-luciferase activity in each sample. Data are the mean relative light unit (RLU; fold induction from luciferase activity in the unstimulated cells) ± SD of triplicates. Statistical differences from the unstimulated control are indicated (^*^ *p*<0.05; ^**^ *p*<0.005). **Panel C.** RAW264.7 cells were stimulated with medium or CpG DNA (6 μg/ml) for the indicated time periods. Messenger RNA levels of *Irak-m* and *β-actin* (loading control) were detected by RT-PCR. All experiments were done more than three times with similar results.

DNA fragments encoding the dominant negative (DN) form of TLR9 (aa 1–873), DN-IRAK2 (aa 1–96), and DN-IRAK4 (aa 1–120) were amplified by PCR using murine cDNA as a template. The resulting DN-TLR9, DN-IRAK2, or DN-IRAK4 encoding cDNA fragments were cloned into pEF6/V5-His-TOPO (Invitrogen). Cloning of DN-IRAK1, DN-MEK1, DN-p38, and DN-JNK1 were previously reported [31]. The CREB-luciferase reporter gene and DN-CREB expression construct pCMV-CREB-S133A were purchased from Clontech (Palo Alto, CA). DN-MyD88 expression construct pIRES2-EGFP-DN-MyD88 was provided by Dr. S.-C. Hong (Indiana Univ., Indianapolis, IN). The IκB-AA expression construct was provided by Dr. G. A. Bishop (University of Iowa, Iowa City, IA). The AP-1-β-galactosidase construct and NF-κB-luciferase construct were provided by Dr. G. Koretzky (University of Pennsylvania, Philadelphia, PA). DNA sequences of all cloned and mutated genes were confirmed by DNA sequencing: analysis and were identical with the previously reported sequences. All PCR primers used for cloning and mutagenesis were purchased from Integrated DNA Technologies, Inc. (Coralville, IA). Sequences of wild type and each modified *cis*-acting element response site in the *Irak-m* promoter region are listed in Table 1.

**Figure 2:**
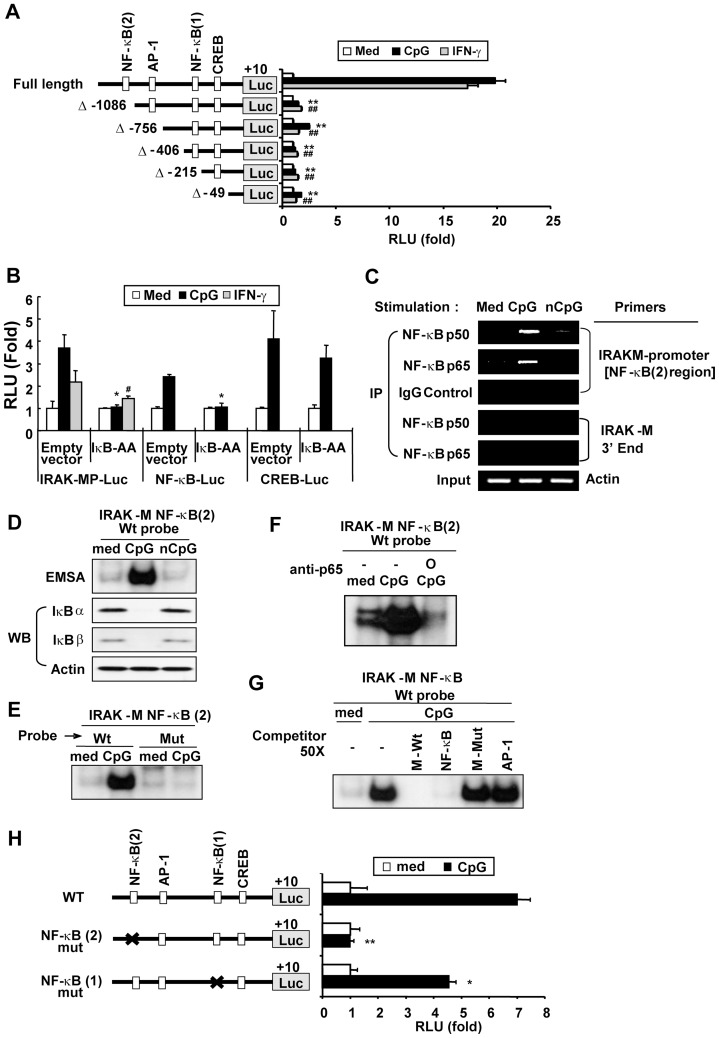
NF-κB is required for CpG DNA-induced *Irak-m* promoter activity. Panel A. RAW264.7 cells were transiently cotransfected with pRL-TK-luciferase and full length (FL) or 5′-deletion mutant (Δ-1086, Δ-756, Δ-406, Δ-215, or Δ-49) *Irak-m* promoter-luciferase reporters and then stimulated with medium, CpG (6 μg/ml), or IFNγ (25 ng/ml) for 24 hr. Luciferase activity in cell extracts was analyzed by the Dual-Luciferase Reporter Assay System and normalized using pRL-TK-luciferase activity in each sample. Data represent the mean RLU (fold induction from luciferase activity of wild type *Irak-m* promoter-luciferase reporter in the unstimulated cells) ± SD of triplicates. Statistical differences from luciferase activity of wild type *Irak-m* promoter-luciferase reporter in the cells stimulated with CpG DNA (^**^ *p*<0.005) or IFNγ (^##^ *p*<0.005) are indicated. **Panel B.** RAW264.7 cells were transiently cotransfected with empty vector or IκB-AA and pRL-TK-luciferase plus *Irak-m*-promoter-luciferase (left section), NF-κB-luciferase (middle section), or CREB-luciferase (right section) reporters. Cells were stimulated with medium, CpG DNA (6 μg/ml), or IFNγ (25 ng/ml) for 36 hr. Luciferase activity in cell extracts was analyzed by the Dual-Luciferase Reporter Assay System and normalized using pRL-TK-luciferase activity in each sample. Data are the mean relative light unit (fold induction from luciferase activity of the indicated reporter in the unstimulated cell+s) ± SD of triplicates. Statistical differences from luciferase activity of the indicated luciferase reporters in the cells transfected with empty vector and stimulated with CpG DNA (^*^ *p*<0.05) or IFNγ (^#^ *p*<0.05) are indicated. **Panel C–G**. RAW264.7 cells were stimulated with medium, CpG DNA (6 μg/ml), or non-CpG DNA (6 μg/ml) for 1 hr. **(C)** To detect NF-κB binding activity to the *Irak-m* promoter region, a ChIP assay was performed with anti-p50, anti-p65, or isotype control IgG Abs. DNA bound to p50 Ab, p65 Ab, or IgG was purified and used as a template for PCR with an *Irak-m* promoter-specific primer set that detects the region containing putative NF-κB (2) consensus site or an *Irak-m*-3′ end-specific primer set. Actin was used as a loading control. IP, immunoprecipitation. (**D**) To detect nuclear DNA binding activity of NF-κB, equal amounts of nuclear extracts (3 μg/lane) were subjected to EMSA using ^32^P-labeled double-stranded ODN containing the NF-κB (2) binding sequences in the *Irak-m* promoter region as a probe. To detect the presence of IκBα and IκBβ, equal amounts of cytoplasmic extract (15 μg/lane) were subjected to SDS-PAGE followed by Western blot analysis. (**E**) Equal amounts of nuclear extracts (3 μg/lane) were subjected to EMSA using ^32^P-labeled double-stranded ODN containing the wild type (Wt) or mutant (Mut) NF-κB (2) binding sequences in the *Irak-m* promoter region as a probe. (**F**) Equal amounts of nuclear extracts (3 μg/lane) were incubated with isotype control IgG or anti-p65 Ab (1 μg) for 30 min at room temperature and then subjected to EMSA using ^32^P-labeled double-stranded ODN containing the NF-κB (2) binding sequences in the *Irak-m* promoter region as a probe. (**G**) Equal amounts of nuclear extracts (3 μg/lane) were incubated with an excess amount (50 X) unlabeled double-stranded ODN containing the wild type (M-Wt) or mutant (M-Mut) NF-κB (2) binding sequences in the *Irak-m* promoter region, universal NF-κB consensus (NF-κB), or universal AP-1 consensus (AP-1) for 30 min at room temperature and then subjected to EMSA using ^32^P-labeled double-stranded ODN containing the NF-κB (2) binding sequences in the *Irak-m* promoter region as a probe. **Panel H.** RAW264.7 cells were transiently cotransfected with full length or site-directed mutants at the NF-κB (2) (−1098/-1088) or NF-κB (1) (−336/−326) sites of *Irak-m*-promoter-luciferase reporters and pRL-TK-luciferase and then stimulated with medium or CpG (6 μg/ml) for 36 hr. Luciferase activity in cell extracts was analyzed by the Dual-Luciferase Reporter Assay System and normalized using pRL-TK-luciferase activity in each sample. Data represent the mean RLU (fold induction from luciferase activity of wild type *Irak-m* promoter-luciferase reporter in the unstimulated cells) ± SD of triplicates. Statistical differences from luciferase activity of wild type *Irak-m* promoter-luciferase reporter in the cells stimulated with CpG DNA are indicated (^*^ *p*<0.05; ^**^ *p*<0.005). All experiments were repeated at least three times with similar results.

### Transfection and reporter gene assays

RAW264.7 cells (2 10^6^ cells/well) were plated into 6-well plates and then incubated for 24 hr to reach approximately 80% confluence. Cells were co-transfected with pRL-TK-luciferase (1 μg) and wild type or mutant *Irak-m* promoter-luciferase reporter constructs (3 μg) using Lipofectamine (Invitrogen) according to the manufacturer's protocol. In some experiments, cells were co-transfected with an equal amount (6 μg) of control empty vector (pEF6/V5-His-TOPO, pIRES-EGFP, pCDNA3, or pUSE), DN-TLR9, DN-MyD88, DN-IRAK1, DN-IRAK2, DN-IRAK4, DN-MEK1, DN-p38, DN-JNK1, IκB-AA, or DN-CREB and reporter gene *Irak-m* promoter-luciferase (3 μg) plus pRL-TK-luciferase (1 μg), NF-κB-luciferase (2 μg) plus pRL-TK-luciferase (1 μg), CREB-luciferase (2 μg) plus pRL-TK-luciferase (1 μg), or AP-1-β-galactosidase (2 μg) for 3 hr. Transfected cells were incubated for 6 hr, pooled, and washed 3 times with culture media. Cells (1 or 2×10^5^ cells/well in 96-well culture plates) were stimulated with medium, CpG DNA (6 μg/ml), non-CpG DNA (6 μg/ml), LPS (50 ng/ml), or IFNγ (25 ng/ml) for designated time periods. In some experiments, transfected cells were pretreated with medium or chloroquine (2.5 μg/ml) for 15 min before stimulation. β-galactosidase and luciferase activities in cell extracts were analyzed according to manufacturers' protocols using the Galacto-Light Plus Reporter gene assay (Tropix, Bedford, MA) and the Dual-Luciferase Reporter Assay System (Promega), respectively. Luciferase activity was normalized using pRL-TK-luciferase activity (*Renilla* luciferase activity) in each sample and expressed as relative light units (RLU). For the AP-1-β-galactosidase assay, equal concentrations of cell lysates were used.

### Western blot assay, electrophoretic mobility shift assay (EMSA), and RT-PCR

Cells were stimulated with medium, CpG DNA (6 μg/ml), or non-CpG DNA (6 μg/ml) for designated time periods. Levels or phosphorylation status of specific proteins in whole cell extracts, DNA binding activities of specific transcription factors in nuclear extracts, and levels of specific gene transcripts in total RNA were analyzed by Western blot assay, EMSA, and RT-PCR, respectively, as described previously [32], [33], [34]. Actin or GAPDH was used as a loading control for all RT-PCR and Western blots. Actin-specific antibody (Ab) was purchased from Santa Cruz Biotechnology, Inc. (Santa Cruz, CA) and all phospho-specific Abs were purchased from Cell Signaling, Inc. (Beverly, MA). Sequences of the ODN probes used for EMSA are previously described [33] or listed in Table 2. The sequences of RT-PCR primers are previously described [19], [30]. All primers were purchased from Integrated DNA Technologies, Inc.

### IL-10-specific enzyme-linked immunosorbent assay (ELISA)

RAW264.7 cells (1×10^6^ cells/ml) were treated with medium or CpG DNA (6 μg/ml) in the presence of various concentrations of cycloheximide (CHX; 0–1 μg/ml) for 24 hr. Culture supernatants were analyzed by ELISA for IL-10 as described previously [35]. Recombinant murine IL-10 and Abs specific for murine IL-10 were purchased from BD PharMingen (San Diego, CA).

### Chromatin immunoprecipitation (ChIP) assay

Cells (1×10^7^) were stimulated with medium, CpG DNA (6 μg/ml), non-CpG DNA (6 μg/ml), or IFNγ (25 ng/ml) for designated time periods and a ChIP assay was performed as previously described [30]. Antibodies specific for NF-κB (anti-p50 and anti-p65) or c-Jun were purchased from Santa Cruz Biotechnology, Inc. Antibody specific for the phosphorylated form of CREB was purchased from Cell Signaling, Inc. ChIP primers designed to amplify the fragment corresponding to the *Irak-m* promoter or the 3′ end of *Irak-m* gene were purchased from Integrated DNA Technologies, Inc. Sequences of the ChIP primers are listed in Table 3.

### Statistical analysis

All experiments were repeated at least three times before analysis. Data are expressed as the mean ± S.D. of triplicates. Two-tailed Student's *t*-test was used to determine statistical significance. Statistical differences with *p*<0.05 and *p*<0.005 are indicated and considered significant.

## Results

### CpG DNA induces Irak-m expression at the transcriptional level without requiring new protein synthesis

Previous studies have shown that TLR ligands, including LPS and CpG DNA, induce expression and production of IRAK-M, a negative regulator in TLR signal transduction, in monocytic cells [20], [30]. However, the mechanism by which TLR ligands induce *Irak-m* expression remains unexplored. Expression of *Irak-m* by TLR ligands can be induced directly through TLR signaling pathways or indirectly by other proteins that are produced by macrophages in response to TLR ligand stimulation. Our study with cycloheximide (CHX), a protein synthesis inhibitor, demonstrated that CpG DNA-mediated induction of *Irak-m* expression does not require new protein synthesis (Fig. S1). This indicates that expression of *Irak-m* induced by TLR9 ligand CpG DNA may be a result of direct signal transduction through a TLR9 signaling pathway.

Expression of *Irak-m* can be regulated by transcriptional and/or post-transcriptional mechanisms. To investigate the mechanism by which CpG DNA induces *Irak-m* expression at the transcriptional level, the proximal 1325 base pairs (bp) in the 5′ region of the mouse *Irak-m* gene were cloned into a luciferase reporter vector. To confirm that the cloned 1325 bp (−1315/+10; translation start site assigned as +1) of the mouse *Irak-m* putative promoter region contains the functional promoter of the *Irak-m* gene, RAW264.7 cells were transiently transfected with this cloned *Irak-m* putative promoter reporter construct (*Irak-m*-promoter-luc) or pGL3 basic vector and then stimulated with CpG DNA for 24 hr. As shown in Figure 1A, CpG DNA induced luciferase activity of the *Irak-m* putative promoter, but not the pGL3 basic vector. As expected, control non-CpG DNA did not induce luciferase activity of the *Irak-m* putative promoter. This result indicates that the cloned proximal 1325 base pairs in the 5′ region of the mouse *Irak-m* gene contain the functional promoter region.

To investigate whether CpG DNA induces *Irak-m*-promoter activity in a time-, dose-, and sequence-dependent manner, RAW264.7 cells were transiently transfected with the *Irak-m*-promoter-luc construct and then stimulated with various concentrations (0.75-12 μg/ml) of CpG DNA or non-CpG DNA for various time periods (6–48 hr). CpG DNA-induced *Irak-m* promoter activity peaked at 24 hr and was evident up to 48 hr after stimulation (Fig. 1B). *Irak-m* message in RAW264.7 cells after CpG DNA stimulation also showed substantial increases with kinetics similar to the putative *Irak-m* promoter activity (Fig. 1C). In addition, CpG DNA induced *Irak-m* promoter-luciferase activity in a dose-dependent manner (Fig. 1D). As expected, control non-CpG DNA failed to induce *Irak-m* promoter-luciferase activity. These results demonstrate that CpG DNA induces transcriptional activity of the *Irak-m* promoter in a time-, dose-, and sequence-dependent manner.

### Activation of NF-κB by CpG DNA is a prerequisite for induction of Irak-m promoter activity

Signal transduction through the TLR9 signaling pathway eventually leads to activation of NF-κB and MAPKs (which lead to activation of AP-1 and CREB) [12], [13], [33], [36], [37]. Sequence analysis revealed that the cloned 1325 base pairs of the 5′-flanking region of the mouse *Irak-m* gene contain putative binding sites for NF-κB (−1098 and -336), AP-1 (−820), and CREB (−138). To identify *cis*-acting elements in the *Irak-m* promoter that are critical for CpG DNA-induced *Irak-m* transcription, we generated a series of deletion mutants of *Irak-m* promoter-luciferase reporter constructs (Fig. 2A). RAW264.7 cells were cotransfected with pRL-TK-luciferase plus full-length, Δ-1086, Δ-756, Δ-406, Δ-215, or Δ-49 *Irak-m* promoter-luciferase reporters. Transfected cells were stimulated with CpG DNA or IFNγ. As shown in Figure 2A, CpG DNA induced an approximately 20-fold increase in the activity of the full-length *Irak-m* promoter-luciferase reporter as compared to the basal unstimulated level. Similar to CpG DNA, IFNγ also induced increases in *Irak-m* promoter luciferase activity. Deletion of a 229 bp region [a region containing an NF-κB (2) site] from the 5′ end of the *Irak-m* promoter region (Δ-1086) ablated *Irak-m* promoter-luciferase activity. Luciferase activity of Δ-1086 in response to CpG DNA or IFNγ was not significantly different from the basal unstimulated *Irak-m* promoter-luciferase activity. Luciferase activities of additional deletion mutants (Δ-756, Δ-406, Δ-215 and Δ-49) were not significantly lower than those of Δ-1086 in the presence or absence of CpG DNA or IFNγ stimulation. These results indicate that the region between –1315 and –1086 bp contains *cis*-acting element(s) required for expression of *Irak-m* and that the NF-κB (2) site may be one of essential *cis*-acting elements regulating transcription of *Irak-m*.

Since our study with *Irak-m* promoter deletion mutant reporters indicated the possible involvement of NF-κB as one of the essential transcription factors that regulate expression of the *Irak-m* gene, we further investigated whether NF-κB is required for transcriptional regulation of CpG DNA-induced *Irak-m* expression. RAW264.7 cells were cotransfected with the *Irak-m* promoter-luc reporter and super-suppressive IκBα (IκB-AA; inhibits activation of NF-κB). Complete suppression of CpG DNA-mediated NF-κB-luciferase activity in cells overexpressing IκB-AA confirmed the functional effectiveness of IκB-AA (Fig. 2B middle section). In contrast, IκB-AA overexpression failed to suppress CREB-luciferase activity induced by CpG DNA stimulation, indicating the specificity of IκB-AA (Fig. 2B, right section). As shown in Figure 2B (left section), *Irak-m* promoter activity in response to CpG DNA was ablated in the RAW264.7 cells by overexpression of IκB-AA. In agreement with this result, various TLR ligands, including CpG DNA (TLR9), LPS (TLR4), and peptidoglycan (TLR2), failed to induce *Irak-m* mRNA expression in RAW264.7 cells in the presence of a pharmacological NF-κB inhibitor (Fig. S2A). To confirm whether the component of the transcription factor NF-κB binds to the NF-κB (2) site in the *Irak-m* promoter region in response to CpG DNA, we performed a ChIP assay using the specific Ab for NF-κB component p50 or p65 and PCR primers specific for the *Irak-m* promoter region containing the NF-κB (2) site. As demonstrated in Figure 2C, CpG DNA, but not control non-CpG DNA, induced increased binding of p65 and p50 in the *Irak-m* promoter region that contains the NF-κB (2) site. As expected, neither p50 nor p65 bound to the 3′ end of the *Irak-m* gene. Non-specific binding of NF-κB components by isotype control IgG was not detected. These results demonstrate that transcription factor NF-κB activated by CpG DNA binds specifically to the promoter region of the *Irak-m* gene. To further determine whether NF-κB actually binds to the predicted *cis*-acting elements present in the *Irak-m* promoter region, an EMSA was performed with the nuclear extracts isolated from RAW264.7 cells stimulated with CpG DNA and a radio-labeled ODN probe containing the predicted distal NF-κB *cis*-acting element [NF-κB (2) (−1098/−1089)] present in the *Irak-m* promoter region. Increased binding of nuclear extracts isolated from CpG DNA-stimulated cells onto the ODN probe that contains the putative NF-κB (2) binding consensus in the *Irak-m* promoter region (−1098/−1089) was detected (Fig. 2D). In contrast, nuclear extracts isolated from medium- or non-CpG DNA-treated cells did not bind to the putative distal NF-κB binding consensus. Nuclear extracts isolated from CpG DNA-stimulated cells did not bind to the ODN probe that contains the mutated *Irak-m* NF-κB (2) binding consensus (Fig. 2E). In addition, nuclear extracts isolated from CpG DNA-stimulated cells failed to bind to the putative distal NF-κB binding consensus in the presence of p65 Ab or unlabeled ODN probes that contain either the putative NF-κB binding consensus of the *Irak-m* promoter region or the common NF-κB binding consensus, while they did bind in the presence of the unlabeled ODN probes that contain the mutated putative *Irak-m* NF-κB (2) binding consensus or the common AP-1 binding consensus (Fig. 2F and 2G). To verify whether NF-κB sites in the *Irak-m* promoter are necessary for CpG DNA-induced *Irak-m* transcription, we generated site-directed point mutation at NF-κB (2) or NF-κB (1) of *Irak-m* promoter-luciferase reporter constructs. RAW264.7 cells were co-transfected with pRL-TK-luciferase plus wild-type *Irak-m* promoter-luc reporter or *Irak-m* promoter-luc reporter with a mutation in either the NF-κB (2) site or NF-κB (1) site. As shown in Figure 2H, CpG DNA failed to induce transcriptional activity of the *Irak-m* promoter-luciferase reporter with a mutation in the NF-κB (2) (−1098/−1089) site. A mutation in the putative NF-κB (1) (−336/−326) site resulted in partially reduced *Irak-m* promoter-luciferase activity in response to CpG DNA (approximately 35% reduction compared to the wild-type *Irak-m* promoter activity). Taken together, our results demonstrated that activation of transcription factor NF-κB and its binding to the consensus site present in the distal region (−1098/−1089) of the *Irak-m* promoter are prerequisite for CpG DNA-mediated *Irak-m* expression and that the proximal NF-κB consensus site (−336/−326) in the *Irak-m* promoter may be dispensable, but still contributes to the optimal induction of *Irak-m* promoter activity by CpG DNA.

### MAPK-mediated activation of AP-1 and CREB contributes to the optimal induction of Irak-m promoter activity

**Figure 3:**
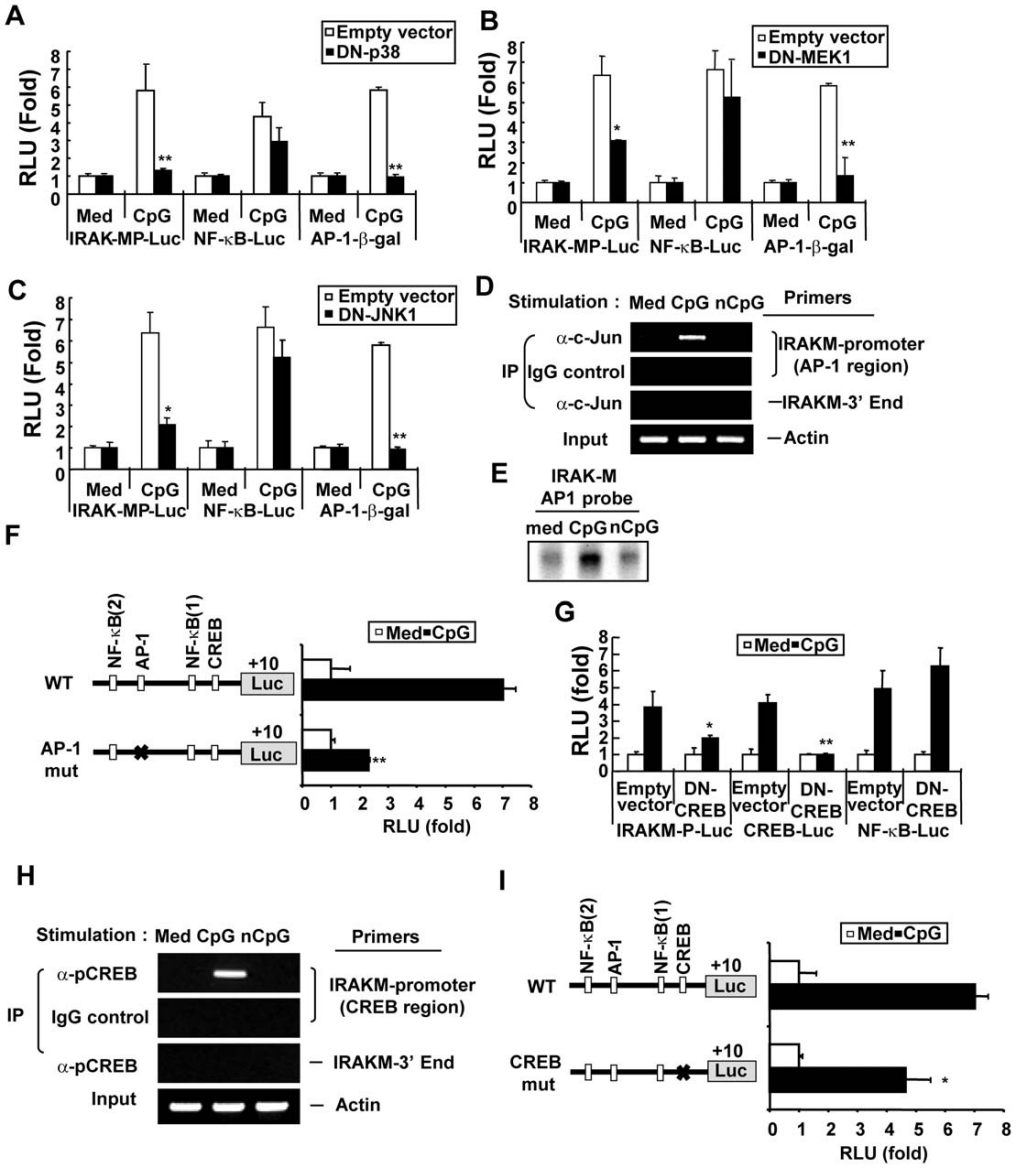
MAPKs, AP-1, and CREB contribute to the optimal induction of *Irak-m* promoter activity. Panels A–C. RAW264.7 cells were cotransfected with *Irak-m*-promoter-luciferase plus pRL-TK-luciferase, NF-κB-luciferase plus pRL-TK-luciferase, or AP-1-β-galactosidase and empty vector or plasmids encoding DN-p38, DN-MEK1, or DN-JNK1. The transfected cells were stimulated with medium or CpG DNA (6 μg/ml). Luciferase activity in cell extracts was analyzed by the Dual-Luciferase Reporter Assay System and normalized using pRL-TK-luciferase activity in each sample. β-galactosidase activity in cell extracts was analyzed using the Galacto-Light Plus Reporter gene assay. Equal concentrations of cell lysates were used for the AP-1-β-galactosidase assay. Data are the mean relative light unit (fold induction from luciferase activity or β-galactosidase activity of the indicated reporter in the unstimulated cells) ± SD of triplicates. Statistical differences from luciferase activity or β-galactosidase activity of the indicated reporters in the cells transfected with empty vector and stimulated with CpG DNA are indicated (^*^ *p*<0.05; ^**^ *p*<0.005). **Panel D**. RAW264.7 cells were stimulated with medium, CpG DNA (6 μg/ml), or non-CpG DNA (6 μg/ml) for 1 hr. To detect AP-1 binding activity to the *Irak-m* promoter region, a ChIP assay was performed with anti-c-Jun Ab or isotype control IgG. DNA bound to c-Jun Ab or IgG was purified and used as a template for PCR with the *Irak-m* promoter-specific primer set that detects *Irak-m* promoter region containing a putative AP-1 binding consensus site or with the *Irak-m*-3′ end-specific primer set. Actin was used as a loading control. IP, immunoprecipitation. **Panel E**. RAW264.7 cells were stimulated with medium, CpG DNA (6 μg/ml), or non-CpG DNA (6 μg/ml) for 1 hr. To detect nuclear DNA binding activity of AP-1, equal amounts of nuclear extracts (3 μg/lane) were subjected to EMSA using ^32^P-labeled double-stranded ODN containing the AP-1 binding consensus sequences in the *Irak-m* promoter region as a probe. **Panel F.** RAW264.7 cells were transiently cotransfected with wild-type or AP-1 (-821/-815) site-mutated (AP-1 mut) *Irak-m*-promoter-luciferase and pRL-TK-luciferase. Cells were stimulated with medium or CpG DNA (6 μg/ml) for 36 hr. Luciferase activity in cell extracts was analyzed by the Dual-Luciferase Reporter Assay System and normalized using pRL-TK-luciferase activity in each sample. Data represent the mean RLU (fold induction from luciferase activity of wild type *Irak-m* promoter-luciferase reporter in the unstimulated cells) ± SD of triplicates. Statistically significant differences from luciferase activity of wild type *Irak-m* promoter-luciferase reporter in the cells stimulated with CpG DNA are indicated (^**^ *p*<0.005). **Panel G.** RAW264.7 cells cotransfected with pRL-TK-luciferase plus *Irak-m*-promoter-luciferase, CREB-luciferase, or NF-κB-luciferase and empty vector or vector encoding DN-CREB were stimulated with medium or CpG DNA (6 μg/ml). Luciferase activity in cell extracts was analyzed by the Dual-Luciferase Reporter Assay System and normalized using pRL-TK-luciferase activity in each sample. Data are the mean relative light unit (fold induction from luciferase activity of the indicated reporter in the unstimulated cells) ± SD of triplicates. Significant differences from luciferase activity of the indicated reporter in the cells transfected with empty vector and stimulated with CpG DNA are indicated (^*^ *p*<0.05; ^**^ *p*<0.005). **Panel H.** RAW264.7 cells were treated with medium, CpG DNA (6 μg/ml), or non-CpG DNA (6 μg/ml) for 1 hr and a ChIP assay was performed with anti-pCREB Ab or isotype control IgG. DNA bound to pCREB Ab or IgG was purified and used as a template for PCR using the *Irak-m* promoter-specific primer set that detects the *Irak-m* promoter region containing a putative CRE consensus site or with the *Irak-m*-3′ end-specific primer set. Actin was used as a loading control. IP, immunoprecipitation. **Panel I.** RAW264.7 cells were transiently cotransfected with full length or CRE (−139/−131) site-mutated (CREB mut) *Irak-m*-promoter-luciferase reporters and pRL-TK-luciferase and then stimulated with medium or CpG DNA (6 μg/ml) for 36 hr. Luciferase activity in cell extracts was analyzed by the Dual-Luciferase Reporter Assay System and normalized using pRL-TK-luciferase activity in each sample. Data represent the mean RLU (fold induction from luciferase activity of wild type *Irak-m* promoter-luciferase reporter in the unstimulated cells) ± SD of triplicates. Statistically significant differences from luciferase activity of wild type *Irak-m* promoter-luciferase reporter in the cells stimulated with CpG DNA are indicated (^*^ *p*<0.05). All experiments were repeated at least three times with similar results.

In addition to the activation of NF-κB, CpG DNA leads to the activation of MAPKs that in turn lead to activation of transcription factors, including AP-1 and CREB [2], [12], [13], [19], [30], [33], [36], [37]. Sequence analysis using the TRASFAC v6.0 revealed that possible binding sites for MAPK-responsive transcription factors, such as AP-1 and CREB, are present in the promoter region of *Irak-m*. Therefore, we investigated whether MAPKs play a functional role in CpG DNA-mediated transcriptional regulation of *Irak-m* expression. RAW264.7 cells were cotransfected with *Irak-m* promoter-luc reporter and expression vectors encoding DN-p38, DN-MEK1, or DN-JNK1. AP-1-β-galactosidase reporter and NF-κB-luciferase reporter were used as positive and negative controls, respectively. AP-1 reporter activity induced by CpG DNA was completely inhibited in DN-p38-, DN-MEK1-, or DN-JNK-overexpressed RAW264.7 cells, indicating that the expressed levels of DN-p38, DN-MEK1, or DN-JNK were sufficient to inhibit the function of CpG DNA-activated p38, MEK1, or JNK, respectively (Fig. 3A, 3B, 3C). In contrast, NF-κB reporter activity induced by CpG DNA was not significantly suppressed by overexpression of DN-p38, DN-MEK1, or DN-JNK, indicating the specificity of DN-p38, DN-MEK1, or DN-JNK (Fig. 3A, 3B, 3C). As demonstrated in Figures 3A, 3B, 3C, CpG DNA-induced *Irak-m* promoter-luciferase activity was significantly reduced by overexpression of DN-p38, DN-MEK1, or DN-JNK. In addition, *Irak-m* mRNA expression in RAW264.7 cells in response to various TLR ligands, including CpG DNA, LPS, and peptidoglycan, was partially inhibited in the presence of a specific pharmacological inhibitor of JNK, p38, or ERK (Fig. S2B). Taken together our results demonstrate that all three MAPKs, which are activated by CpG DNA, contribute to *Irak-m* transcription.

Because our results showed that MAPKs play a functional role in CpG DNA-induced *Irak-m* transcription, and the *Irak-m* promoter region contains consensus binding sites for MAPK-dependent transcription factors AP-1 and CREB, we further investigated whether AP-1 and/or CREB are required for transcriptional regulation of CpG DNA-induced Irak-m expression. To determine whether the component of the transcription factor AP-1 binds to the *Irak-m* promoter region in response to CpG DNA, we performed a ChIP assay using the AP-1 component c-Jun-specific Ab and the *Irak-m* promoter AP-1 region-specific PCR primers. Of note, we previously reported that c-Jun is one of the components in the AP-1 complex activated by CpG DNA [38]. As shown in Figure 3D, CpG DNA, but not control non-CpG DNA, induced increased binding of c-Jun in the *Irak-m* promoter region, demonstrating that transcription factor AP-1 activated by CpG DNA binds to the promoter region of the *Irak-m* gene. To further determine whether AP-1 components actually bind to the predicted *cis*-acting elements present in the *Irak-m* promoter region, an EMSA was performed with the nuclear extracts isolated from RAW264.7 cells stimulated with CpG DNA and a radio-labeled ODN probe containing the predicted AP-1 *cis*-acting element (−820/−815) present in the *Irak-m* promoter region. Increased binding of nuclear extracts isolated from CpG DNA-stimulated cells onto the ODN probe that contains the putative AP-1 binding consensus in the *Irak-m* promoter region was detected (Fig. 3E). In contrast, nuclear extracts isolated from medium- or non-CpG DNA-treated cells did not bind to the putative AP-1 binding consensus. To verify whether the AP-1 site in the *Irak-m* promoter has a role in CpG DNA-induced *Irak-*m transcription, we generated site-directed point mutation at the AP-1 site (−820/−815) of *Irak-m* promoter-luciferase reporter constructs. RAW264.7 cells were co-transfected with pRL-TK-luciferase plus wild-type *Irak-m* promoter-luc reporter or *Irak-m* promoter-luc reporter with a mutation in the AP-1 site. As shown in Figure 3F, a mutation in the putative AP-1 site reduced CpG DNA-induced *Irak-m* promoter-luciferase activity to approximately 32% of the wild-type *Irak-m* promoter activity. These results demonstrate that CpG DNA activates AP-1 that binds to the consensus site in the *Irak-m* promoter and that AP-1 contributes to the optimal induction of *Irak-m* promoter activity.

It has previously been demonstrated that p38 activated by CpG DNA leads to the activation of transcription factor CREB [12], and a putative CREB-binding site is present in the *Irak-m* promoter region. To investigate whether CREB plays a role in CpG DNA-induced *Irak-m* promoter activity, RAW264.7 cells were co-transfected with the *Irak-m* promoter-luc reporter and the DN-CREB expression vector. Overexpression of DN-CREB partially, but significantly, inhibited CpG DNA-induced *Irak-m* promoter-luciferase activity (Fig. 3G). As expected, overexpression of DN-CREB completely suppressed CpG DNA-induced CREB-luciferase activity without affecting the CpG DNA-induced NF-κB-luciferase activity, confirming the specificity and functional activity of DN-CREB. To investigate whether the activated CREB binds to the *Irak-m* promoter region in response to CpG DNA, we performed a ChIP assay using the specific Ab for the phosphorylated form of CREB (pCREB) and PCR primers specific for the *Irak-m* promoter region containing a putative CRE consensus site. CpG DNA, but not control non-CpG DNA, induced increased binding of pCREB in the *Irak-m* promoter region, demonstrating that transcription factor CREB activated by CpG DNA binds to the promoter region of the *Irak-m* gene (Fig. 3H). To further confirm the requirement of CREB for CpG DNA-induced *Irak-m* promoter activity, we modified the CRE (−138/−131) consensus site in the *Irak-m* promoter region using site-directed mutagenesis. As shown in Figure 3I, mutation of the CREB-binding consensus site resulted in partial but significant reduction in *Irak-m* promoter-luciferase activity in response to CpG DNA stimulation. These results demonstrate that CREB plays a functional role in CpG DNA-mediated *Irak-m* expression. Our results demonstrate that AP-1 and CRE are functional *cis*-acting elements in the *Irak-m* promoter region, and that in addition to NF-κB, transcription factors AP-1 and CREB also contribute to the optimal induction of *Irak-m* promoter activity in response to CpG DNA.

### In addition to IRAK4 and IRAK1, IRAK2 and PKD1 play a pivotal role in CpG DNA-mediated Irak-m expression

**Figure 4:**
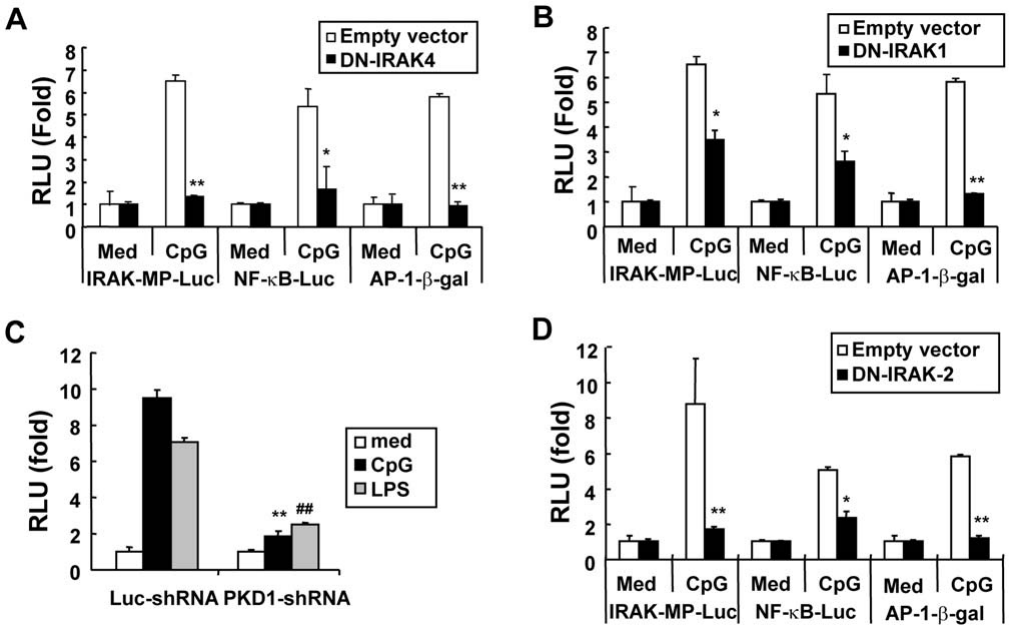
CpG DNA-mediated induction of *Irak-m* promoter activity is dependent on IRAK2 and PKD1 as well as IRAK4 and IRAK1. Panels A, B, and D. RAW264.7 cells were transiently cotransfected with empty vector or plasmids encoding DN-IRAK4 (A), DN-IRAK1 (B), or DN-IRAK2 (D) and *Irak-m*-promoter-luciferase plus pRL-TK-luciferase reporters, NF-κB-luciferase plus pRL-TK-luciferase reporters, or AP-1-β-galactosidase reporter. Cells were stimulated with medium or CpG DNA (6 μg/ml). Luciferase activity in cell extracts was analyzed by the Dual-Luciferase Reporter Assay System and normalized using pRL-TK-luciferase activity in each sample. β-galactosidase activity in equal amounts of cell extracts was analyzed using the Galacto-Light Plus Reporter gene assay. Data are the mean relative light unit (fold induction from luciferase activity or β-galactosidase activity of the indicated reporter in the unstimulated cells) ± SD of triplicates. Significant differences from luciferase activity or β-galactosidase activity of the indicated reporter in the cells transfected with empty vector and stimulated with CpG DNA are indicated (^*^ *p*<0.05; ^**^ *p*<0.005). **Panel C**. Control luciferase-knockdown macrophages (Luc-shRNA) or *Prkd1*-knockdown macrophages (PKD-1shRNA) were cotransfected with *Irak-m*-promoter-luciferase and pRL-TK-luciferase. Transfected cells were treated with medium, CpG DNA (6 µg/ml), or LPS (50 ng/ml) for 36 hr. Luciferase activity in cell extracts was analyzed by the Dual-Luciferase Reporter Assay System and normalized using pRL-TK-luciferase activity in each sample. Data are the mean relative light unit (fold induction from luciferase activity of unstimulated cells) ± SD of triplicates. Significant differences from luciferase activity in Luc-shRNA cells stimulated with CpG DNA (^**^ *p*<0.005) or LPS (^##^ *p*<0.005) are indicated. All experiments were repeated at least three timeswith similar results.

It has previously been demonstrated that CpG DNA interacts with its receptor TLR9 in an endosomal compartment [31], [36], [39], and all known biologic effects of TLR9 are dependent on its signaling adaptor molecule MyD88 [2], [20]. We also found that CpG DNA induces *Irak-m* promoter activity through an endosomal pH-sensitive TLR9/MyD88-dependent pathway (Fig. S3). Binding of MyD88 to TLR9 leads to the sequential recruitment and activation of IRAK family proteins (IRAK4 and IRAK1), PKD1, and TRAF6, which in turn leads to activation of upstream modulators in NF-κB and MAPKs activation pathways [1], [2], [18], [19]. To determine whether IRAK4 and/or IRAK1 contributes to CpG DNA-mediated induction of *Irak-m* transcription, RAW264.7 cells were transiently co-transfected with *Irak-m*-promoter-luc reporter vector and vector expressing DN-IRAK4 or DN-IRAK1. As shown in Figure 4A, overexpression of DN-IRAK4 ablated CpG DNA-mediated induction of *Irak-m* promoter activity, as well as transcriptional activity of NF-κB and AP-1. These results indicate that IRAK4 is required for CpG DNA-induced *Irak-m* transcription. Overexpression of DN-IRAK1 resulted in partial but significant inhibition of CpG DNA-induced *Irak-m* promoter activity (Fig. 4B). As reported previously [31], CpG DNA-induced transcriptional activity of NF-κB was also partially inhibited in RAW264.7 cells overexpressing DN-IRAK1. However, overexpression of DN-IRAK1 completely abolished transcriptional activity of AP-1 induced by CpG DNA (Fig. 4B). Increased concentrations of DN-IRAK1 did not further inhibit CpG DNA-mediated induction of *Irak-m* promoter activity (data not shown), indicating that overexpression of DN-IRAK1 was sufficient to inhibit the function of CpG DNA and that incomplete inhibition of *Irak-m* promoter activities by DN-IRAK1 was not due to an ineffective dominant negative function. Taken together, these results suggest that *Irak-m* expression induced by CpG DNA may require additional signaling modulators downstream of IRAK4, or the function of IRAK1 in TLR9 signaling for *Irak-m* expression may be supplemented by other signaling modulator(s).

Recent studies demonstrated that a serine/threonine kinase, PKD1, is recruited to the TLR9-receptor signaling complex and physically interacts with IRAK4, IRAK1, and TRAF6 upon CpG DNA stimulation [19]. Activation of PKD1 by CpG DNA is dependent on IRAK4 and IRAK1, while it is independent of TRAF6 [18]. In addition, PKD1 is required for the CpG DNA-mediated TRAF6 ubiquitination and TAK1 activation, which leads to the activation of NF-κB and MAPK and subsequent gene expression [18], [19]. If an additional signaling modulator that regulates *Irak-m* expression downstream of IRAK4, but not downstream of IRAK1, is present, the contribution of PKD1 to CpG DNA-mediated induction of *Irak-m* transcription may be partial, as seen with IRAK1, because activation of PKD1 by CpG DNA is dependent on IRAK1. Therefore, we further investigated whether PKD1 contributes to CpG DNA-mediated induction of *Irak-m* transcription. Our studies with a pharmacological PKD/PKC inhibitor, Gö6976, indicated that TLR ligands fail to induce expression of *Irak-m* in RAW264.7 cells when TLR-mediated PKD1 activation is suppressed (Fig. S2C). To confirm this finding with a genetic approach, control luciferase-knockdown macrophages and PKD1 gene (*Prkd1*)-knockdown macrophages were transiently transfected with *Irak-m* promoter-luc reporter. As demonstrated in Figure 4C, *Irak-m* promoter activity was increased by CpG DNA or LPS in control luciferase-knockdown macrophages. However, CpG DNA and LPS failed to induce *Irak-m* promoter activity in *Prkd1*-knockdown macrophages. Neither expression of TLR9 signaling molecules (including TLR9, MyD88, IRAK4, IRAK1, IRAK2, and TRAF6) nor biologic response to other stimuli (such as IFNγ) was suppressed in *Prkd1*-knockdown macrophages compared to those in control luciferase-knockdown macrophages [18], [19]. Of note, knockdown of PKD3, a PKD protein family member that is not involved in TLR signaling, did not alter levels of *Irak-m* mRNA expression induced in response to various TLR ligands (Fig. S4). These results demonstrate that PKD1 is required for expression of *Irak-m* induced by CpG DNA. Our results also suggest a possibility that the function of IRAK1 in TLR9 signaling for *Irak-m* expression (and also for PKD1 activation) may be supplemented or compensated for by other signaling modulator(s).

A recent study has demonstrated that although it is dispensable for activation of the initial TLR signaling cascade, IRAK2 is activated by IRAK4 in the absence of IRAK1 and is essential for sustaining TLR-induced activation of NF-κB and expression of genes encoding certain cytokines [16]. We also found that although TLR9-mediated activation of MAPKs and NF-κB at the early phase is ablated in macrophages that lack IRAK1, their activation at the late phase is not inhibited, indicating that there is a signaling modulator that replaces the function of IRAK1 in the late phase of TLR9 signal transduction (Fig. S5). Considering that *Irak-m* is a late-response gene, these observations suggest a possibility that expression of *Irak-m* by CpG DNA may require IRAK2, and IRAK2 may be a signaling molecule that supplements IRAK1 at the late phase of TLR9 signal transduction. To investigate whether IRAK2 is essential for CpG DNA-mediated induction of *Irak-m* transcription, RAW264.7 cells were transiently co-transfected with *Irak-m*-promoter-luc reporter and control empty vector or DN-IRAK2. As shown in Figure 4D, CpG DNA-mediated induction of transcriptional activity of the *Irak-m* promoter was completely inhibited in RAW264.7 cells overexpressing DN-IRAK2. In addition, CpG DNA-induced transcriptional activity of AP-1 was ablated by overexpression of DN-IRAK2. CpG DNA-induced transcriptional activity of NF-κB was also significantly inhibited by overexpression of DN-IRAK2. These results demonstrate that IRAK2 is required for induction of *Irak-m* promoter activity by CpG DNA stimulation and suggest that IRAK2 might be the signaling modulator that supplements or substitutes for IRAK1 in induction of *Irak-m* gene expression in TLR9 signaling. These results demonstrate that IRAK4, IRAK1, IRAK2, and PKD1 are essential for CpG DNA-induced *Irak-m* transcription, and suggest that IRAK2 may be the additional factor in TLR9 signaling that can supplement or compensate for the function of IRAK1 in CpG DNA-mediated *Irak-m* expression.

### IRAK2 contributes to Irak-m expression through sustaining activation of PKD1, NF-κB and MAPKs

**Figure 5:**
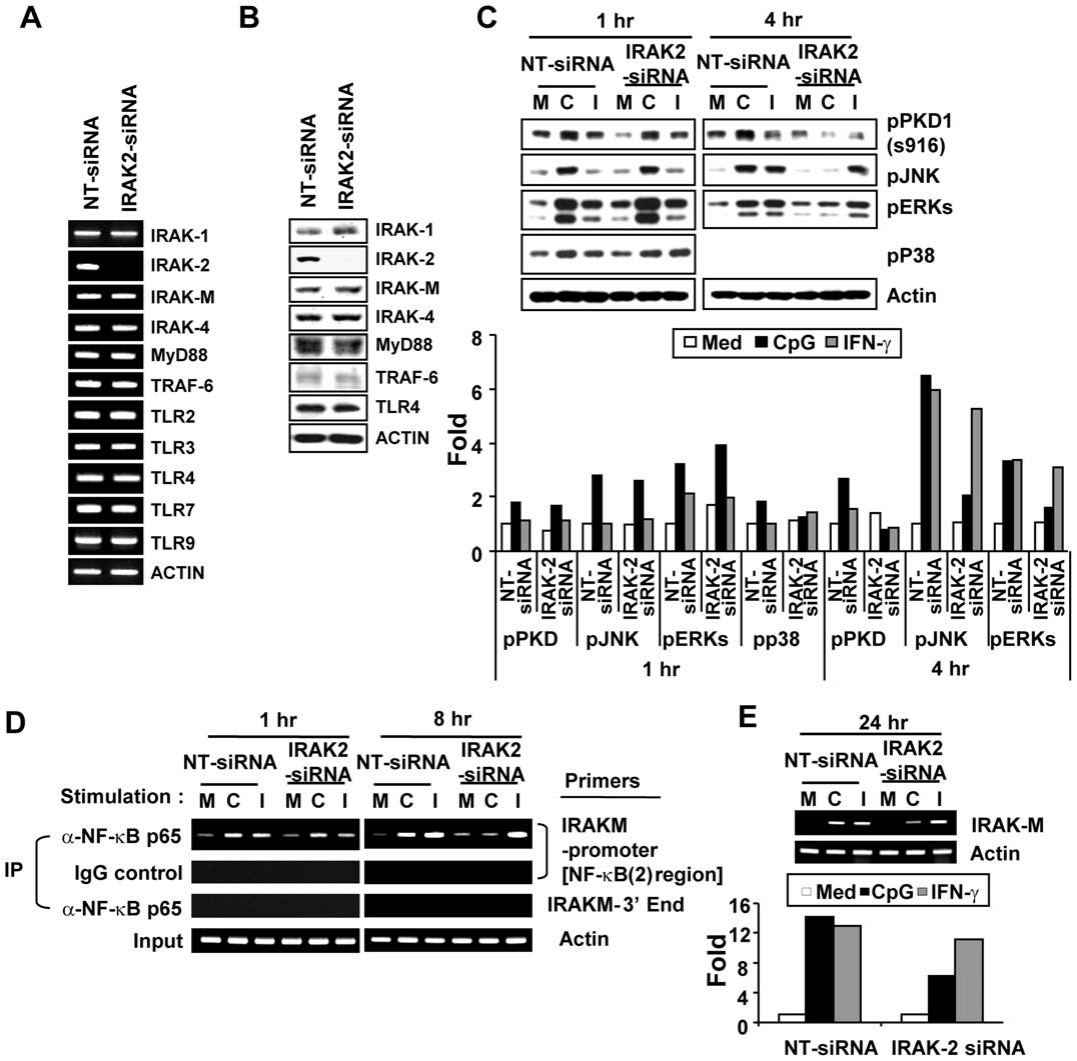
IRAK2 is necessary for sustaining activation of PKD1, MAPKs, and NF-κB after stimulation by CpG DNA. RAW264.7 cells were transiently transfected with non-target siRNA (NT siRNA; control) or *Irak2*-siRNA (*Irak2*-knockdown) using lipofectamine. **Panel A.** Messenger RNA levels of the indicated genes were analyzed by RT-PCR. **Panel B.** Levels of the indicated proteins were analyzed using Western blot assay. **Panels C – E.** Control or *Irak2*-knockdown cells were stimulated with medium (M), CpG DNA (6 μg/ml; C), or IFNγ (25 ng/ml; I) for the indicated time periods. (**C, top**) The activation status of PKD1 and MAPKs was detected by phospho-specific Western blot assay. (**C, bottom**) Quantitation of *panel C top* by densitometry. The density of each protein band was quantitated by densitometry and normalized to the density of the actin band in the same sample. Data represent the fold induction from the normalized densitometric value of each protein band of the unstimulated NT-siRNA control sample. **(D)** To detect NF-κB binding activity to the *Irak-m* promoter region, a ChIP assay was performed with anti-p65 Ab or isotype control IgG. DNA bound to p65 Ab or IgG was purified and used as a template for PCR with the *Irak-m* promoter-specific primer set that detects the *Irak-m* promoter region containing the putative NF-κB (2) consensus site or the *Irak-m*-3′ end-specific primer set. Actin was used as a loading control. IP, immunoprecipitation. (**E, top**) Total RNA was extracted and RT-PCR for *Irak-m* was performed. Actin was used as a loading control. (**E, bottom**) Quantitation of *panel E top* by densitometry. The density of *Irak-m* mRNA band was quantitated by densitometry and normalized to the density of the actin band in the same sample. Data represent the fold induction from the normalized densitometric value of *Irak-m* mRNA band of the unstimulated NT-siRNA control sample. Data represent results obtained from three separate experiments.

Because our findings support a possibility that IRAK2 may be a signaling modulator that compensates for the function of IRAK1 in the TLR9 signaling pathway at the late phase when IRAK1 is not available, we further investigated whether IRAK2 is involved in regulation of CpG DNA-mediated induction of *Irak-m* expression by contributing to the sustained activation of one or more downstream signaling modulators and/or transcription factors using *Irak2*-knockdown cells. Control (NT-siRNA) and *Irak2*-knockdown (*Irak2*-siRNA) macrophages were generated by transiently transfecting RAW264.7 cells with non-target siRNA and *Irak2*-specific siRNA, respectively. Expression of *Irak2* mRNA and protein was almost completely inhibited in *Irak2*-knockdown cells (Fig. 5A and 5B). In contrast, mRNA and protein levels of other genes tested in *Irak2*-knockdown cells were comparable to those in the control macrophages. These results demonstrate that *Irak2*-siRNA specifically and effectively silenced *Irak2* expression. Control and *Irak2*-knockdown macrophages were stimulated with medium, CpG DNA, or IFNγ and then activation of PKD1, MAPKs, and NF-κB and expression of *Irak-m* mRNA at early and late time points were assessed. Activation of PKD1 and MAPKs (JNK, ERK, and p38) at 1 hr by CpG DNA stimulation was not impaired in *Irak2*-knockdown macrophages, indicating that IRAK2 is dispensable for the initial phase activation of these signaling modulators by CpG DNA. In contrast, activation of PKD1, JNK, and ERK at 4 hr after CpG DNA stimulation was almost completely impaired in *Irak2*-knockdown macrophages (Fig. 5C). Activation of p38 at 4 hr after CpG DNA stimulation was not detected in either control macrophages or *Irak2*-knockdown macrophages. Of note, IFNγ-mediated activation of JNK and ERK was not impaired in *Irak2*-knockdown macrophages. These results indicate that IRAK2 is essential for sustaining activation of PKD1 and MAPKs in response to CpG DNA stimulation. Since NF-κB is the transcription factor absolutely required for *Irak-m* expression and CpG DNA-mediated NF-κB activation is dependent on PKD1 [18], [19], [20], we further investigated whether IRAK2 actually contributes to expression of *Irak-m* by sustaining activation of NF-κB. Alterations in the binding activity of NF-κB to the *Irak-m* promoter region in response to CpG DNA in *Irak2*-knockdown macrophages was assessed using a ChIP assay. As shown in Figure 5D, CpG DNA induced increased binding of NF-κB component p65 to the *Irak-m* promoter region in control macrophages at 1 hr and at 8 hr after CpG DNA stimulation. The level of binding of the NF-κB component p65 to the *Irak-m* promoter region at 1 hr after CpG DNA stimulation in *Irak2*-knockdown macrophages was comparable to that in control macrophages. However, CpG DNA failed to induce binding of p65 to the *Irak-m* promoter region in *Irak2*-knockdown macrophages at 8 hr after CpG DNA stimulation. In addition, *Irak-m* mRNA expression in response to CpG DNA was substantially suppressed in the *Irak2*-knockdown macrophages (Fig. 5E). These results indicate that sustained activation of NF-κB mediated through an IRAK2-dependent manner was necessary for *Irak-m* expression. Of note, neither binding of p65 to the *Irak-m* promoter region nor *Irak-m* mRNA expression induced by IFNγ was affected by *Irak2*-knockdown. Our results provide direct evidence that IRAK2 is essential for CpG DNA-induced *Irak-m* transcription through sustained activation of TLR9/MyD88 downstream signaling modulators and transcription factors, including PKD1, MAPKs, and NF-κB, and suggest that IRAK2 may be an additional factor in TLR9 signaling that can replace the function of IRAK1.

## Discussion

IRAK-M, a pseudoenzyme unlike other IRAK family proteins, is expressed mainly in monocytic cells in response to stimulation with various TLR ligands *in vivo* and *in vitro* [20], [30], [40]. IRAK-M inhibits MyD88-dependent TLR signaling by preventing dissociation of IRAK1 and IRAK4 from MyD88 and formation of the IRAK1/TRAF6 complex [20]. As a result, IRAK-M contributes to the attenuation of inflammatory gene expression. Although the biochemical mechanisms by which IRAK-M blocks the TLR signaling have been revealed and the induction of IRAK-M expression by TLR ligands has been observed, it is currently unknown how TLR ligand stimulation results in expression of IRAK-M. In the current study, we have demonstrated a novel regulatory function of IRAK2 and PKD1 in the transcription of *Irak-m*. We found that the up-regulation of *Irak-m* expression by TLR9 is controlled at the transcriptional level through multiple transcription factors, including NF-κB, AP-1, and CREB. Among the *cis*-acting elements present in the *Irak-m* promoter region, the distal NF-κB binding site (−1098/−1089) is the most critical for *Irak-m* transcription. The critical role of NF-κB in *Irak-m* transcription was supported by results showing complete inhibition of CpG DNA-mediated *Irak-m* promoter activity by overexpression of IκB-AA and *Irak-m* mRNA expression by a pharmacological inhibitor of NF-κB. Deletion or point mutation of the distal NF-κB binding site in the *Irak-m* promoter region results in ablated *Irak-m* promoter activity, indicating the absolute requirement of this site for *Irak-m* expression. ChIP assay and EMSA demonstrated that the majority of NF-κB components p65 and p50 bind to the distal NF-κB binding site rather than to the proximal site (−336/−326) (data not shown). Accordingly, the contribution of the proximal NF-κB binding site (−336/−326) is minimal and dispensable for *Irak-m* transcription. In addition to NF-κB, MAPK-dependent transcription factors AP-1 and CREB (although they are dispensable), contribute to the optimal induction of *Irak-m* promoter activity by CpG DNA through binding to the AP-1 and CRE consensus sites, respectively, present in the *Irak-m* promoter region. The roles of transcription factors AP-1 and CREB and their upstream regulator MAPKs in the optimal expression of *Irak-m* were further supported by results showing partial inhibition of TLR ligand-mediated *Irak-m* mRNA expression by pharmacological inhibitors of MPAKs. All these transcription factors are also known to be involved in regulation of expression of numerous early responsive proinflammatory genes by CpG DNA and other TLR ligands [2], [12], [13], [37], [41], [42]. We did not find any specific transcription factor that is unique to the expression of *Irak-m* in response to TLR9 ligand CpG DNA.

Although the same transcription factors are involved in expression of genes with different regulatory roles, it is possible that there are other unique mechanisms that differentiate expression of early responsive genes (and/or proinflammatory genes, such as *tnf*) *vs* expression of late responsive genes (and/or negative regulatory genes, such as *Irak-m*). Although *Irak-m* is one of the late responsive genes, expression of *Irak-m* in macrophages in response to TLR ligands, including CpG DNA, does not require new protein synthesis. Rather, it appears to be directly regulated by the proximal TLR signaling events. As expected, CpG DNA-induced expression of the *Irak-m* gene and protein is dependent on an endosomal acidification, TLR9, MyD88, and IRAK4. IRAK1, which interacts with MyD88 and IRAK4 and is activated by IRAK4, has been shown to be indispensable for the early phase activation of PKD1 [18], [19]. In addition, the transcriptional activity of AP-1-responsive promoter in macrophages and type I IFN expression in pDCs in response to CpG DNA are absolutely dependent on IRAK1 [31], [43], [44]. In contrast to these early signaling events and early gene expression, the contribution of IRAK1 to transcriptional activity of the *Irak-m* promoter in response to TLR9 ligand was only partial. Similar to the partial regulatory effects of IRAK1 on TLR9-mediated transcription of *Irak-m*, previous studies have demonstrated that CpG DNA-mediated induction of transcriptional activity of NF-κB-responsive promoter and cyclooxygenase (*Cox*) *2*-promoter is only partially dependent on IRAK1 [31]. In addition, fibroblasts isolated from *Irak1* ^-/-^ mice or *Irak1*-knockdown macrophages show dramatically diminished, but not completely abolished, activation of NF-κB, p38, ERK, and JNK, and production of proinflammatory cytokines TNFα, IL-6 and IFNγ in response to ligands of TLR/IL-1R family members [30], [45], [46]. These findings suggest the possibilities that either IRAK1 is one of the diverging points in the TLR9 signaling pathway or there is some other factor(s) that might supplement the function of IRAK1 in TLR9-mediated expression of certain genes, including *Irak-m* and *Cox2*.

Recent studies indicate that PKD1 is recruited to the TLR/MyD88 receptor complex *via* an interaction with IRAK4, IRAK1, and TRAF6 and is activated by TLR ligands [18], [19]. MyD88-dependent activation of MAPKs and NF-κB and expression of proinflammatory genes in response to ligands of the TLR/IL-1R family members are dependent on PKD1. It has been shown previously that TLR ligands are unable to activate PKD1 in *Irak1*-knockdown macrophages [18]. However, unlike the partial effects of IRAK1 on *Irak-m* expression, our results with *Prkd1*-knockdown macrophages demonstrated that PKD1 is indispensable for CpG DNA-induced *Irak-m* expression. Because ligands of the TLR/IL-1R family members induce sustained activation of MAPKs and NF-κB, and PKD1 is required for MyD88-dependent activation of MAPKs and NF-κB, it is possible that TLR/IL-1R ligands induce sustained activation of PKD1. The function of IRAK1 at the later phase of TLR/IL-1R signal transduction in sustaining activation of PKD1 may be compensated for by other yet to be identified signaling modulator(s). These findings further support the possibility that there is some other signaling modulator(s) that is utilized by the TLR9 (and other TLR/IL-1R) signaling pathway to compensate for the function of IRAK1 in sustaining activation of PKD1, MAPKs and NF-κB and expression of certain late phase genes, such as *Irak-m*.

Possible redundancy among IRAK family members has been suggested, and IRAK2 appears to have a function similar to that of IRAK1. Demonstration that the forced overexpression of IRAK2 bypasses the upstream receptor signaling and results in increased *Irak-m* transcription would provide more direct evidence that it directly and transcriptionally regulates *Irak-m* gene expression. However, to the best of our knowledge, there is no published evidence that overexpression of IRAK2 spontaneously induces activation of itself and its downstream events and expression of its target genes without signal transduction initiated by TLRs. Although complete understanding of the role of IRAK2 in the TLR/IL-1R signaling pathway awaits further intensive investigation, several recent studies have revealed that, like IRAK1, IRAK2 interacts with and is phosphorylated by IRAK4 and is a functional kinase [16], [17], [47]. *Irak2*-deficent mice are resistant to LPS-induced septic shock due to the impaired production of proinflammatory cytokines and chemokines, indicating a critical role for IRAK2 in the TLR4-mediated proinflammatory response [48]. In addition, overexpression of IRAK2 in Irak1-deficient cells is sufficient to restore responsiveness to IL-1 [49]. Furthermore, the kinase activity of IRAK2 is sustained for longer than that of IRAK1 after TLR ligand stimulation. IRAK2 is critical for sustaining activation of NF-κB and p38 at the late phase of TLR/IL-1R signal transduction, when IRAK1 is not available, indicating that both IRAK1 and IRAK2 regulate inflammatory responses through the kinase activity of IRAK1 followed by IRAK2 [16]. In agreement with these previous findings, our results also demonstrated that although IRAK2 does not affect TLR9-mediated activation of PKD1, MAPKs, and NF-κB at the initial phase of TLR9 signaling, it is indispensable for sustaining activation of these signaling modulators in response to TLR9 ligand stimulation. In addition, IRAK2 is absolutely required for induction of *Irak-m* promoter activity and AP-1 and NF-κB transcriptional activity by CpG DNA. Our results further support the previously suggested possibility that IRAK2 is the protein that replaces the function of IRAK1 in the later phase of TLR signaling, when IRAK1 has disappeared after the initial phase of activation.

In summary, we demonstrate that induction of *Irak-m* expression by TLR9 ligand CpG DNA stimulation does not require new protein synthesis and is directly regulated at the transcriptional level through the TLR9 signaling pathway. In addition to MyD88, IRAK4, and IRAK1, IRAK2 and PKD1 are critical for *Irak-m* transcription. Sustained activation of NF-κB is essential for *Irak-m* expression, and IRAK2 contributes to *Irak-m* expression by replacing the function of IRAK1 in activation of signaling modulators and transcription factors, including PKD1 and NF-κB, at the late phase of TLR9 signal transduction, when IRAK1 is not available.

## Supporting Information

Figure S1 **Induction of** ***Irak-m*** **expression by CpG DNA does not require new protein synthesis.** RAW264.7 cells were stimulated with medium or CpG DNA (6 μg/ml) in the presence of various concentrations (0–1 μg/ml) of cycloheximide (CHX), a protein synthesis inhibitor, for 24 hr. **Panel A.** Production of IL-10 protein in response to CpG DNA was used as a positive control to monitor efficacy of CHX and analyzed by ELISA. Data are the mean (pg/ml) ± SD of triplicates. Statistical differences from CpG DNA-stimulated control group are indicated (^**^ *p*<0.005). **Panel B.** Messenger RNA levels of *Irak-m* and *β-actin* (loading control) were detected by RT-PCR. **Panel C.** Cell viability was measured using trypan blue vital staining. Data represent mean % of viable cells ± S.D. of triplicates. N.D.  =  Not Done. All experiments were done more than three times with similar results. Our results demonstrated that CpG DNA failed to induce IL-10 production in the presence of CHX, confirming the inhibitory effect of CHX on new protein synthesis. In contrast, CpG DNA up-regulated *Irak-m* mRNA expression even in the presence of CHX, indicating that new protein synthesis is not required for CpG DNA-mediated induction of *Irak-m* expression.
(DOC)

Figure S2 **Effects of pharmacological inhibitors of NF-κB, MAPKs, or PKD/PKC on TLR ligand-mediated** ***Irak-m*** **expression.** RAW264.7 cells were stimulated with medium, CpG DNA (6 μg/ml; TLR9 ligand), LPS (50 ng/ml; TLR4 ligand) or peptidoglycan (5 μg/ml; PGN; TLR2 ligand) for 24 hr in the presence or absence of vehicle (DMSO), Bay11–7082 (10 μM; NF-κB inhibitor), U0126 (1.25 μM; ERK inhibitor), SB203580 (2.5 μM; p38 inhibitor), SP600125 (5 μM; JNK inhibitor), Gö6976 (500 ng/ml; PKD/PKC inhibitor) or Gö6983 (500 ng/ml; PKC inhibitor). Messenger RNA levels of *Irak-m* and *β-actin* (loading control) were detected by RT-PCR. Panel B bottom is quantitation of *panel B top* by densitometry. The density of *Irak-m* mRNA band was quantitated by densitometry and normalized to the density of the actin band in the same sample. Data represent the fold induction from the normalized densitometric value of *Iram-m* mRNA band of the unstimulated control sample. All experiments were repeated at least three times with similar results. *Irak-m* mRNA expression induced in response to various TLR ligands (CpG DNA, LPS, and PGN) was almost completely ablated in RAW264.7 cells pre-treated with NF-κB inhibitor Bay11-7082 or PKD/PKC inhibitor Gö6976. In contrast, PKC inhibitor Gö6983 failed to inhibit TLR-mediated *Irak-m* mRNA expression. These data indicate that NF-κB and PKD family proteins, probably PKD1, play an indispensable role in TLR ligand-mediated *Irak-m* expression. *Irak-m* mRNA expression induced in response to various TLR ligands in RAW264.7 cells pre-treated with U0126, SB203580, or SP600125 was only partially suppressed, indicating that MAPKs (ERK, p38, and JNK) may be dispensable for expression of *Irak-m*, but they contribute to the optimal expression of *Irak-m*.
(DOC)

Figure S3 **CpG DNA induces** ***Irak-m*** **promoter activity through an endosomal pH-sensitive TLR9/MyD88-dependent pathway.** **Panel A.** RAW264.7 cells were transiently cotransfected with *Irak-m*-promoter-luciferase and pRL-TK-luciferase reporters. Cells were pretreated with medium or chloroquine (2.5 μg/ml; inhibitor of endosomal acidification) for 15 min and then stimulated with medium, CpG DNA (6 μg/ml; TLR9 ligand), or LPS (50 ng/ml; TLR4 ligand; used as a negative control) for 24 hr. **Panels B–C.** RAW264.7 cells were transiently cotransfected with *Irak-m*-promoter-luciferase reporter plus pRL-TK-luciferase and empty vector or plasmids encoding DN-TLR9 (TIR domain deleted form of TLR9) or DN-MyD88 (death domain deleted form of MyD88). Cells were stimulated with medium or CpG DNA (6 μg/ml). Luciferase activity in cell extracts was analyzed by the Dual-Luciferase Reporter Assay System and normalized using pRL-TK-luciferase activity in each sample. Data present the mean relative luciferase unit (fold induction from luciferase activity in the unstimulated cells) ± SD of triplicates. Statistical differences from luciferase activity in the cells transfected with empty vector and stimulated with CpG DNA are indicated (^**^ *p*<0.005). **Panel D.** Peritoneal macrophages isolated from wild-type, *Tlr9* ^−/−^ or *Myd88* ^−/−^ mice were stimulated with medium, CpG DNA (6 μg/ml), LPS (50 ng/ml; used as a negative control) or IFNγ (25 ng/ml; used as a negative control) for 24 hr. Messenger RNA levels of *Irak-m* and *β-actin* (loading control) were detected by RT-PCR. All experiments were repeated at least three times with similar results. It has previously been demonstrated that CpG DNA is endocytosed by leukocytes and interacts with its receptor TLR9 in an endosomal compartment [31], [36], [39], and all known biologic effects of TLR9 have been shown to be dependent on its signaling adaptor molecule, MyD88 [2], [20]. Therefore, we investigated whether CpG DNA-induced *Irak-m* expression is mediated through an endosomal pH-sensitive TLR9/MyD88-dependent signaling pathway. Chloroquine (inhibitor of endosomal acidification) did not suppress LPS-induced *Irak-m*-promoter-luciferase activity, indicating that chloroquine at the concentration used is not toxic. In contrast, CpG DNA-induced *Irak-m*-promoter-luciferase activity was completely abolished in the presence of chloroquine, indicating that CpG DNA induces *Irak-m* expression through an endosomal pH-sensitive pathway. In addition, CpG DNA-mediated induction of *Irak-m* promoter activity was ablated in RAW264.7 cells overexpressing either DN-TLR9 or DN-MyD88. Similarly, CpG DNA failed to induce expression of *Irak-m* message in peritoneal macrophages isolated from either *Tlr9* ^−/−^ or *Myd88* ^−/−^ mice. Of note, levels of LPS-mediated *Irak-m* expression in *Tlr9* ^−/−^ macrophages and levels of IFNγ-mediated *Irak-m* expression in *Myd88* ^−/−^ macrophages were comparable to levels of *Irak-m* expression induced by LPS and IFNγ, respectively, in wild-type macrophages. Taken together, our results demonstrated that CpG DNA induces *Irak-m* promoter activity through an endosomal pH-sensitive TLR9/MyD88-dependent pathway.
(DOC)

Figure S4 **TLR ligand-mediated** ***Irak-m*** **mRNA expression is not altered in** ***Prkd3*** **-knockdown macrophages.** RAW264.7 cells were transiently transfected with non-target siRNA (NT siRNA; control) or *Prkd3*-siRNA (*Prkd3*-knockdown) using lipofectamine. **Panel A.** Messenger RNA levels of the indicated genes were analyzed by RT-PCR. **Panel B.** Control or *Prkd3*-knockdown cells were stimulated with medium (M), CpG DNA (6 μg/ml; C), LPS (50 ng/ml; L) or PGN (5 μg/ml; P) for 45 min. The activation status of PKD1 and MAPKs was detected by phospho-specific Western blot assay. Degradation of IκBα was detected by Western blot assay. **Panel C.** Control or *Prkd3*-knockdown cells were stimulated with medium (M), CpG DNA (6 μg/ml; C), LPS (50 ng/ml; L) or PGN (5 μg/ml; P) for 24 hr. Messenger RNA levels of *Irak-m* and *β-actin* (loading control) were detected by RT-PCR. Expression of *Prkd3* mRNA was completely silenced in *Prkd3*-knockdown cells. In contrast, mRNA levels of other genes tested in *Prkd3*-knockdown cells were comparable to those in the control macrophages. These results demonstrate that *Prkd3*-siRNA specifically and effectively silenced *Prkd3* expression. Activation of NF-κB (judged by phosphorylation of IκBα) and MAPKs (JNK, ERK, and p38) by TLR ligands was not impaired in *Prkd3*-knockdown macrophages, indicating that PKD3 does not play a role in the activation of these signaling modulators by TLR ligands. In addition, levels of expression of *Irak-m* mRNA induced by TLR ligand stimulation in *Prkd3*-knockdown macrophages was comparable to those in control macrophages, demonstrating that PKD3 is not involved in TLR-induced expression of *Irak-m*.
(DOC)

Figure S5 **IRAK1 is dispensable for sustaining activation of MAPKs and NF-κB after stimulation by CpG DNA.** Control luciferase-knockdown macrophages (Luc-shRNA) or IRAK1-knockdown macrophages (IRAK1-shRNA) [30] were stimulated with medium or CpG DNA (6 μg/ml) for the indicated time periods. **Panel A.** The activation status of MAPKs was detected by phospho-specific Western blot assay. Actin was used as a loading control. **Panel B.** To detect nuclear DNA binding activity of NF-κB, equal amounts of nuclear extracts (3 μg/lane) were subjected to EMSA using ^32^P-labeled double-stranded ODN containing the NF-κB binding consensus sequences as a probe. Data represent results obtained from three separate experiments. CpG DNA failed to activate JNK, ERK, and p38 MAPKs within 1 hr in IRAK1-knockdown macrophages. However, activation of JNK and ERK at the sustaining phase (4–8 hr after the CpG DNA stimulation) was not suppressed in IRAK1-knockdown macrophages. Of note, activation of p38 at the sustaining phase was not detected in either control or IRAK1-knockdown macrophages. Activation of NF-κB at the early phase in TLR9 signal transduction (within 1 hr after CpG DNA stimulation) was substantially reduced in IRAK1-knockdown macrophages compared to that in control macrophages. In contrast, activation of NF-κB at the late phase (4–8 hr after the CpG DNA stimulation) in IRAK1-knockdown macrophages was comparable with that in control macrophages. These results indicate that IRAK1 is essential for activation of MAPKs and NF-κB at the early phase but dispensable at the sustained phase of TLR9 signal transduction, and that there might be another signaling modulator that substitutes for the function of IRAK1 in the late phase of TLR9 signaling.
(DOC)
