# Supplementary figures and images for: Interleukin-1 Receptor-Associated Kinase 2- and Protein Kinase D1-Dependent Regulation of IRAK-Monocyte Expression by CpG DNA
Source: PLoS One. 2012 Aug 23;7(8):e43970. doi: 10.1371/journal.pone.0043970 (PMC3426515; doi:10.1371/journal.pone.0043970)

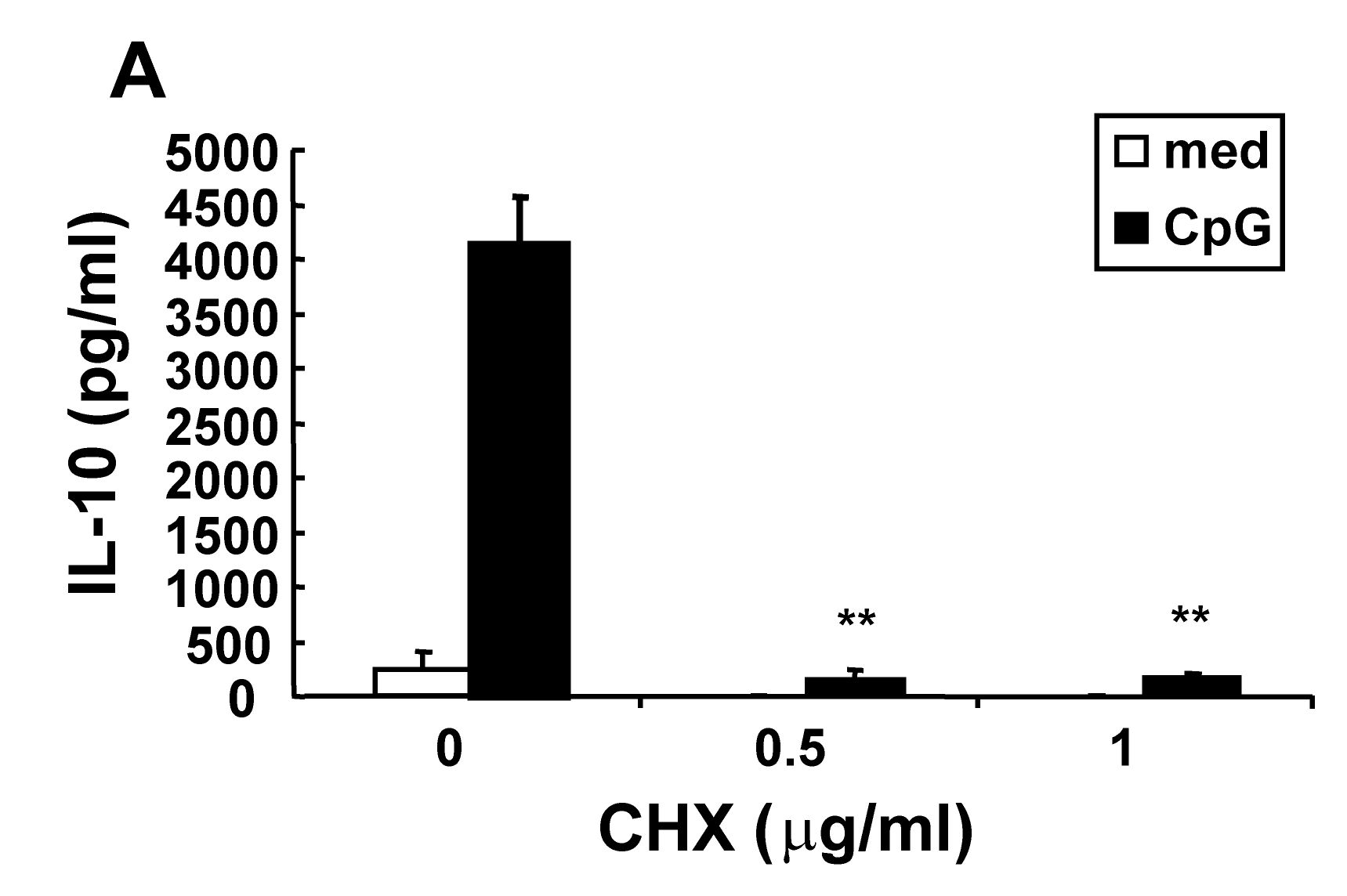

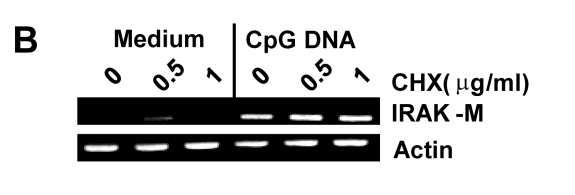

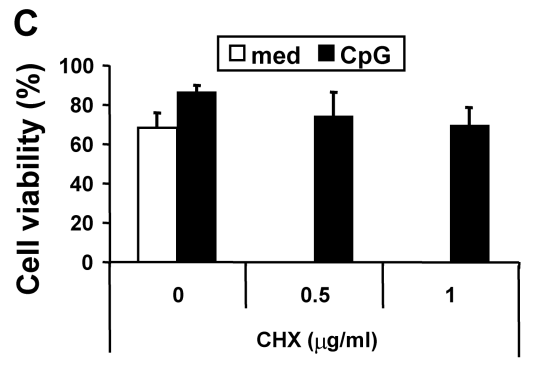

Supplement: Figure S1 — Induction of Irak-m expression by CpG DNA does not require new protein synthesis. RAW264.7 cells were stimulated with medium or CpG DNA (6 μg/ml) in the presence of various concentrations (0–1 μg/ml) of cycloheximide (CHX), a protein synthesis inhibitor, for 24 hr. Panel A. Production of IL-10 protein in response to CpG DNA was used as a positive control to monitor efficacy of CHX and analyzed by ELISA. Data are the mean (pg/ml) ± SD of triplicates. Statistical differences from CpG DNA-stimulated control group are indicated (** p<0.005). Panel B. Messenger RNA levels of Irak-m and β-actin (loading control) were detected by RT-PCR. Panel C. Cell viability was measured using trypan blue vital staining. Data represent mean % of viable cells ± S.D. of triplicates. N.D. = Not Done. All experiments were done more than three times with similar results. Our results demonstrated that CpG DNA failed to induce IL-10 production in the presence of CHX, confirming the inhibitory effect of CHX on new protein synthesis. In contrast, CpG DNA up-regulated Irak-m mRNA expression even in the presence of CHX, indicating that new protein synthesis is not required for CpG DNA-mediated induction of Irak-m expression. (DOC) [file pone.0043970.s001.doc]

**
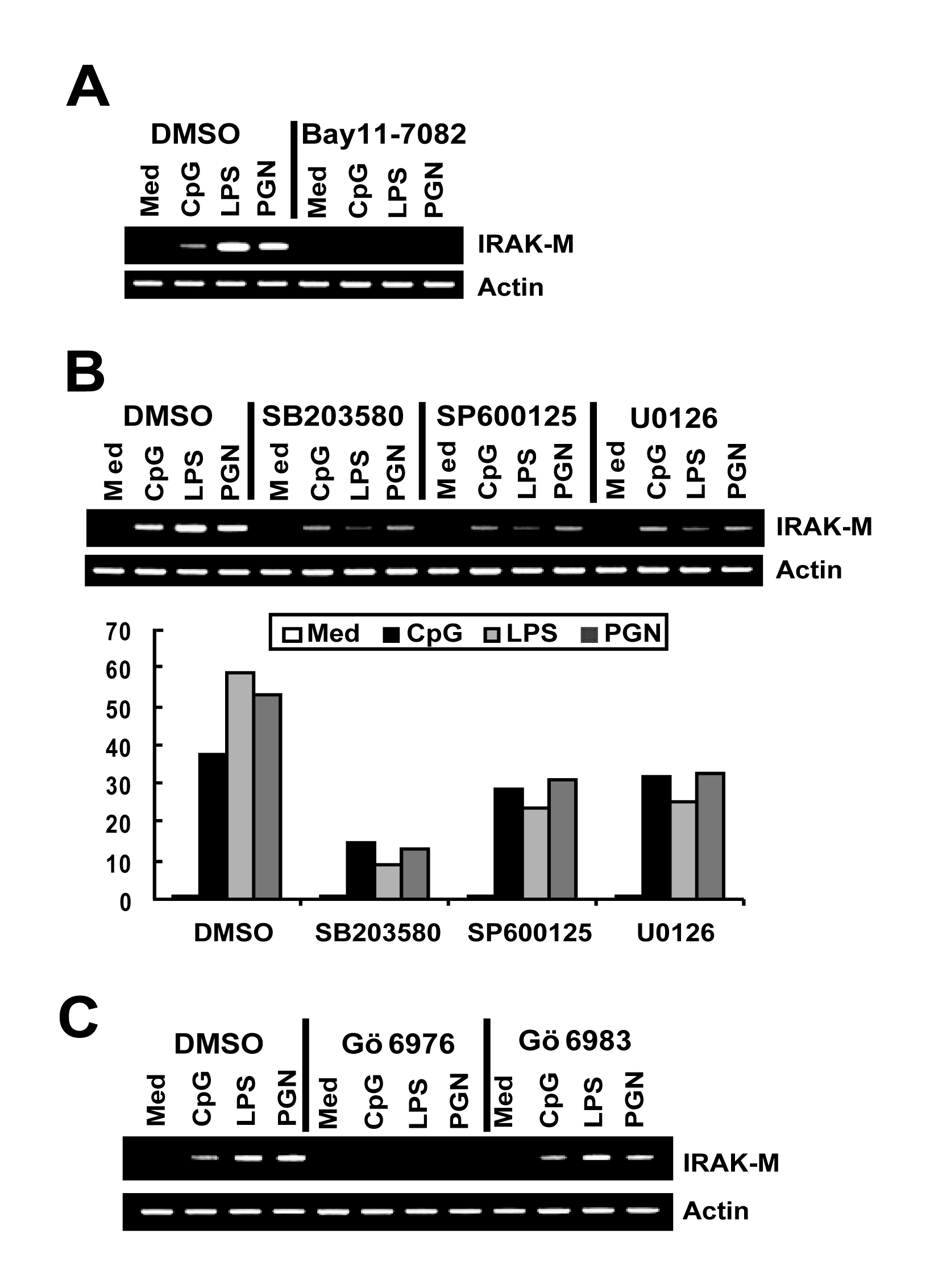
**

Supplement: Figure S2 — Effects of pharmacological inhibitors of NF-κB, MAPKs, or PKD/PKC on TLR ligand-mediated Irak-m expression. RAW264.7 cells were stimulated with medium, CpG DNA (6 μg/ml; TLR9 ligand), LPS (50 ng/ml; TLR4 ligand) or peptidoglycan (5 μg/ml; PGN; TLR2 ligand) for 24 hr in the presence or absence of vehicle (DMSO), Bay11–7082 (10 μM; NF-κB inhibitor), U0126 (1.25 μM; ERK inhibitor), SB203580 (2.5 μM; p38 inhibitor), SP600125 (5 μM; JNK inhibitor), Gö6976 (500 ng/ml; PKD/PKC inhibitor) or Gö6983 (500 ng/ml; PKC inhibitor). Messenger RNA levels of Irak-m and β-actin (loading control) were detected by RT-PCR. Panel B bottom is quantitation of panel B top by densitometry. The density of Irak-m mRNA band was quantitated by densitometry and normalized to the density of the actin band in the same sample. Data represent the fold induction from the normalized densitometric value of Iram-m mRNA band of the unstimulated control sample. All experiments were repeated at least three times with similar results. Irak-m mRNA expression induced in response to various TLR ligands (CpG DNA, LPS, and PGN) was almost completely ablated in RAW264.7 cells pre-treated with NF-κB inhibitor Bay11-7082 or PKD/PKC inhibitor Gö6976. In contrast, PKC inhibitor Gö6983 failed to inhibit TLR-mediated Irak-m mRNA expression. These data indicate that NF-κB and PKD family proteins, probably PKD1, play an indispensable role in TLR ligand-mediated Irak-m expression. Irak-m mRNA expression induced in response to various TLR ligands in RAW264.7 cells pre-treated with U0126, SB203580, or SP600125 was only partially suppressed, indicating that MAPKs (ERK, p38, and JNK) may be dispensable for expression of Irak-m, but they contribute to the optimal expression of Irak-m. (DOC) [file pone.0043970.s002.doc]

**
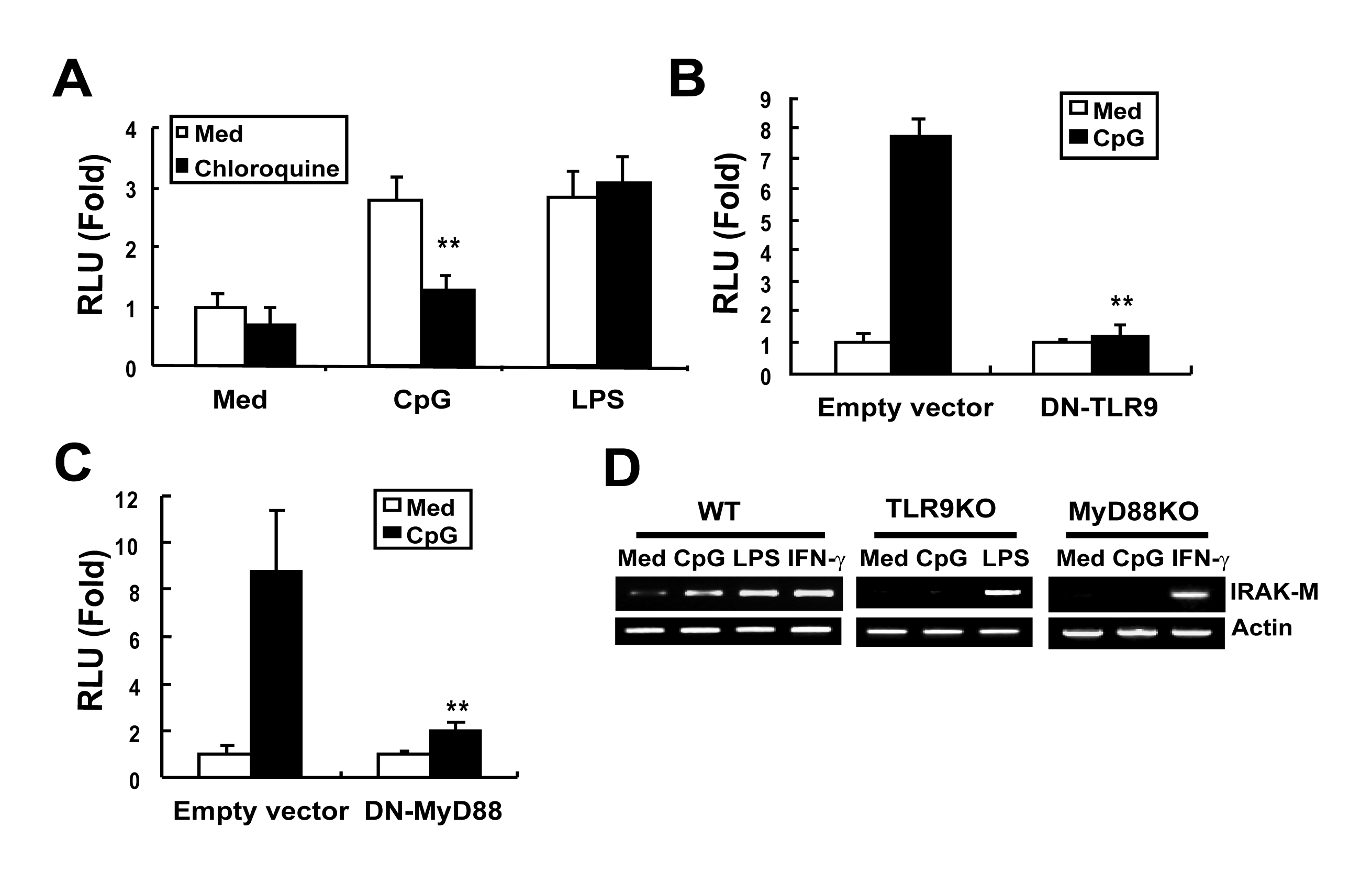
**

Supplement: Figure S3 — CpG DNA induces Irak-m promoter activity through an endosomal pH-sensitive TLR9/MyD88-dependent pathway. Panel A. RAW264.7 cells were transiently cotransfected with Irak-m-promoter-luciferase and pRL-TK-luciferase reporters. Cells were pretreated with medium or chloroquine (2.5 μg/ml; inhibitor of endosomal acidification) for 15 min and then stimulated with medium, CpG DNA (6 μg/ml; TLR9 ligand), or LPS (50 ng/ml; TLR4 ligand; used as a negative control) for 24 hr. Panels B–C. RAW264.7 cells were transiently cotransfected with Irak-m-promoter-luciferase reporter plus pRL-TK-luciferase and empty vector or plasmids encoding DN-TLR9 (TIR domain deleted form of TLR9) or DN-MyD88 (death domain deleted form of MyD88). Cells were stimulated with medium or CpG DNA (6 μg/ml). Luciferase activity in cell extracts was analyzed by the Dual-Luciferase Reporter Assay System and normalized using pRL-TK-luciferase activity in each sample. Data present the mean relative luciferase unit (fold induction from luciferase activity in the unstimulated cells) ± SD of triplicates. Statistical differences from luciferase activity in the cells transfected with empty vector and stimulated with CpG DNA are indicated (** p<0.005). Panel D. Peritoneal macrophages isolated from wild-type, Tlr9 −/− or Myd88 −/− mice were stimulated with medium, CpG DNA (6 μg/ml), LPS (50 ng/ml; used as a negative control) or IFNγ (25 ng/ml; used as a negative control) for 24 hr. Messenger RNA levels of Irak-m and β-actin (loading control) were detected by RT-PCR. All experiments were repeated at least three times with similar results. It has previously been demonstrated that CpG DNA is endocytosed by leukocytes and interacts with its receptor TLR9 in an endosomal compartment [31], [36], [39], and all known biologic effects of TLR9 have been shown to be dependent on its signaling adaptor molecule, MyD88 [2], [20]. Therefore, we investigated whether CpG DNA-induced Irak-m expression is mediated through an endosomal p [file pone.0043970.s003.doc]

**
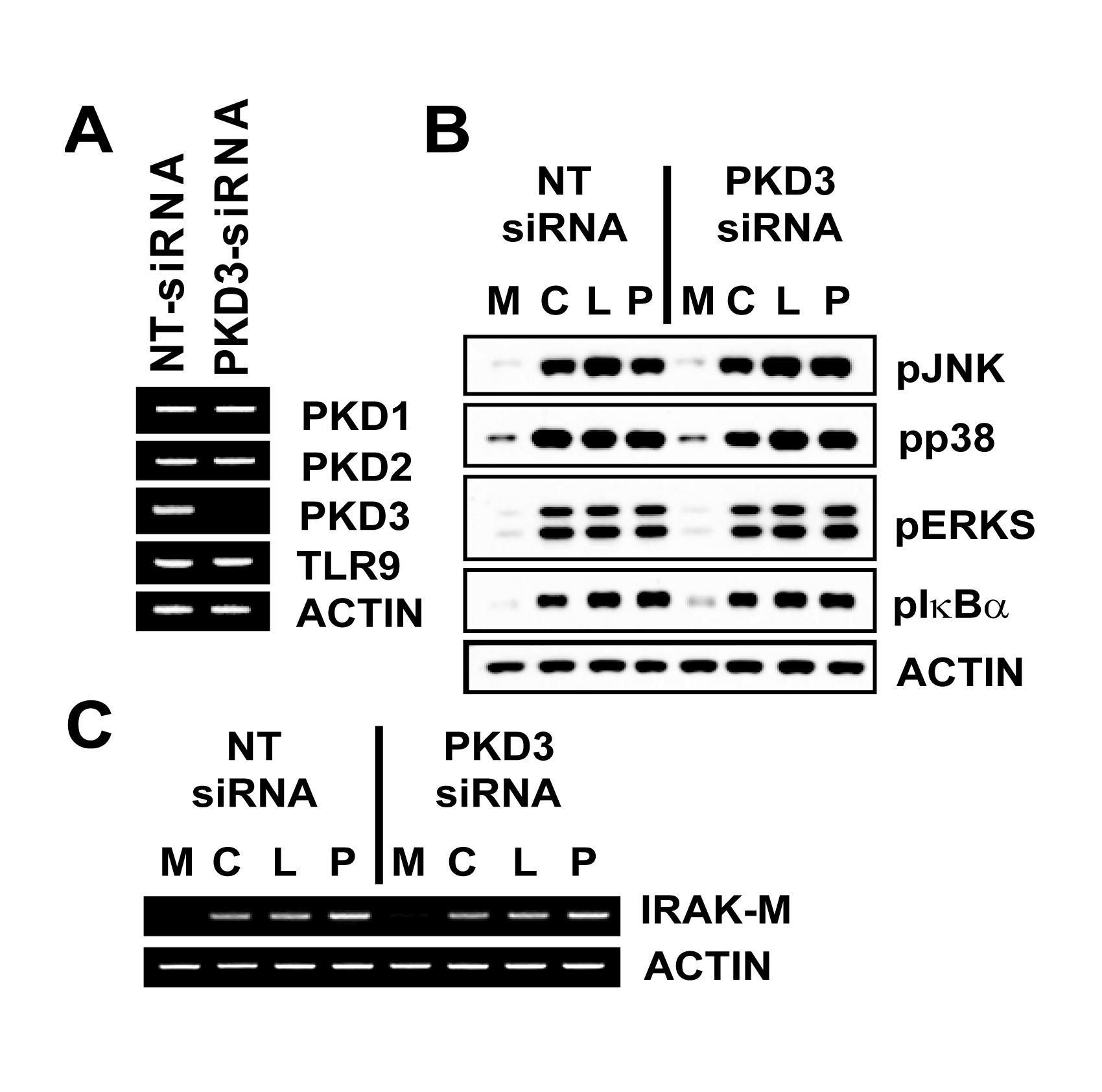
**

Supplement: Figure S4 — TLR ligand-mediated Irak-m mRNA expression is not altered in Prkd3 -knockdown macrophages. RAW264.7 cells were transiently transfected with non-target siRNA (NT siRNA; control) or Prkd3-siRNA (Prkd3-knockdown) using lipofectamine. Panel A. Messenger RNA levels of the indicated genes were analyzed by RT-PCR. Panel B. Control or Prkd3-knockdown cells were stimulated with medium (M), CpG DNA (6 μg/ml; C), LPS (50 ng/ml; L) or PGN (5 μg/ml; P) for 45 min. The activation status of PKD1 and MAPKs was detected by phospho-specific Western blot assay. Degradation of IκBα was detected by Western blot assay. Panel C. Control or Prkd3-knockdown cells were stimulated with medium (M), CpG DNA (6 μg/ml; C), LPS (50 ng/ml; L) or PGN (5 μg/ml; P) for 24 hr. Messenger RNA levels of Irak-m and β-actin (loading control) were detected by RT-PCR. Expression of Prkd3 mRNA was completely silenced in Prkd3-knockdown cells. In contrast, mRNA levels of other genes tested in Prkd3-knockdown cells were comparable to those in the control macrophages. These results demonstrate that Prkd3-siRNA specifically and effectively silenced Prkd3 expression. Activation of NF-κB (judged by phosphorylation of IκBα) and MAPKs (JNK, ERK, and p38) by TLR ligands was not impaired in Prkd3-knockdown macrophages, indicating that PKD3 does not play a role in the activation of these signaling modulators by TLR ligands. In addition, levels of expression of Irak-m mRNA induced by TLR ligand stimulation in Prkd3-knockdown macrophages was comparable to those in control macrophages, demonstrating that PKD3 is not involved in TLR-induced expression of Irak-m. (DOC) [file pone.0043970.s004.doc]

**
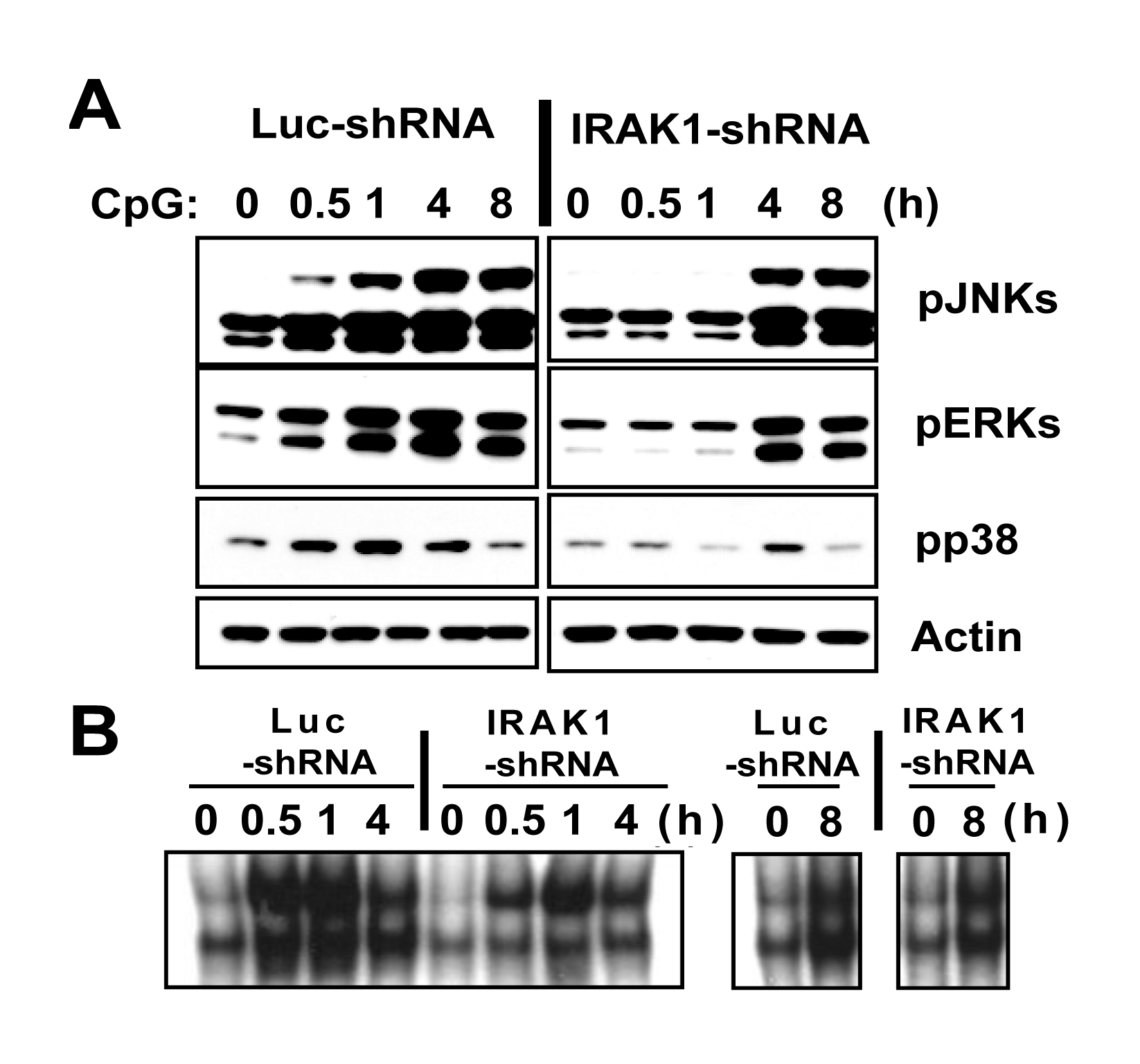
**

Supplement: Figure S5 — IRAK1 is dispensable for sustaining activation of MAPKs and NF-κB after stimulation by CpG DNA. Control luciferase-knockdown macrophages (Luc-shRNA) or IRAK1-knockdown macrophages (IRAK1-shRNA) [30] were stimulated with medium or CpG DNA (6 μg/ml) for the indicated time periods. Panel A. The activation status of MAPKs was detected by phospho-specific Western blot assay. Actin was used as a loading control. Panel B. To detect nuclear DNA binding activity of NF-κB, equal amounts of nuclear extracts (3 μg/lane) were subjected to EMSA using 32P-labeled double-stranded ODN containing the NF-κB binding consensus sequences as a probe. Data represent results obtained from three separate experiments. CpG DNA failed to activate JNK, ERK, and p38 MAPKs within 1 hr in IRAK1-knockdown macrophages. However, activation of JNK and ERK at the sustaining phase (4–8 hr after the CpG DNA stimulation) was not suppressed in IRAK1-knockdown macrophages. Of note, activation of p38 at the sustaining phase was not detected in either control or IRAK1-knockdown macrophages. Activation of NF-κB at the early phase in TLR9 signal transduction (within 1 hr after CpG DNA stimulation) was substantially reduced in IRAK1-knockdown macrophages compared to that in control macrophages. In contrast, activation of NF-κB at the late phase (4–8 hr after the CpG DNA stimulation) in IRAK1-knockdown macrophages was comparable with that in control macrophages. These results indicate that IRAK1 is essential for activation of MAPKs and NF-κB at the early phase but dispensable at the sustained phase of TLR9 signal transduction, and that there might be another signaling modulator that substitutes for the function of IRAK1 in the late phase of TLR9 signaling. (DOC) [file pone.0043970.s005.doc]
